# A Facile Strategy for the High Yielding, Quantitative Conversion of Polyglycol End-Groups to Amines

**DOI:** 10.3390/polym13091403

**Published:** 2021-04-26

**Authors:** Jie Yan, Paula Facal Marina, Anton Blencowe

**Affiliations:** Applied Chemistry and Translational Biomaterials (ACTB) Group, Clinical and Health Sciences, University of South Australia, Adelaide, SA 5001, Australia; jie.yan@mymail.unisa.edu.au (J.Y.); paula.facalmarina@unisa.edu.au (P.F.M.)

**Keywords:** polyglycol, amino end-group, Zn-mediated reduction, poly(ethylene glycol), end-group transformations

## Abstract

Amino end-group functionalised polyglycols are important intermediates in the synthesis of sophisticated polymeric architectures and biomaterials. Herein, we report a facile strategy for the end-group conversion of hydroxyl-terminated polyglycols to amino-terminated polyglycols in high isolated yields and with excellent end-group fidelity. Following traditional conversion of polyglycol hydroxyl end-groups to azides via the corresponding mesylate, reduction with zinc in the presence of ammonium chloride afforded a range of amino end-group functionalised poly(ethylene glycol) and poly(propylene glycol) homopolymers and copolymers with isolated yields of 82–99% and end-group conversions of >99% as determined by NMR spectroscopy and MALDI ToF MS. Furthermore, this process is applicable to a sequential reagent addition approach without intermediate polymer isolation steps with only a slight reduction in yield and end-group conversion (95%). Importantly, a simple work-up procedure provides access to high purity polyglycols without contamination from other reagents.

## 1. Introduction

Polyglycols, and in particular poly(ethylene glycol) (PEG), are widely applied in the synthesis of polymeric delivery systems and biomaterials due to their good biocompatibility, low protein fouling and low immunogenicity [1,2,3,4]. In recent years, this has been somewhat accelerated by the increased commercial availability of homo- and hetero-functionalised PEG derivatives from specialist companies, albeit at significant cost compared to more conventional PEG derivatives. For bioconjugation studies, amine functionalised derivatives feature prominently due to their cross-reactivity with bioactives [5,6,7], and amine terminated PEGs have been used extensively when conjugating polymers to therapeutics (e.g., drugs and peptides), targeting ligands and imaging agents [8,9,10,11]. Therefore, the efficient introduction of amine groups to the end of polyglycols is important for their application to conjugates, delivery systems, biomaterials and block copolymer synthesis [8,11,12,13,14,15,16,17].

The cost of amino-terminated PEGs can be prohibitively expensive compared to their hydroxy-terminated counterparts, which may make them inaccessible for some laboratories, or limit large-scale synthesis and use. For the purpose of obtaining larger quantities of amino-terminated polyglycols, we screened published methods describing their synthesis from hydroxyl-terminated polyglycols [18,19,20,21,22,23,24,25,26,27,28,29,30,31,32], for which five approaches have been mainly reported and employed. Several groups have employed a two-step strategy involving the initial conversion of PEG hydroxyl end-groups to phthalimido end-groups via the Mitsunobu reaction followed by the addition of hydrazine to afford amino-terminated PEGs with >98% end-group conversion and isolated yields of 75–88% [20,23,24]. The method originally reported by Bückmann et al. involving the conversion of PEG hydroxyl end-groups to halide end-groups with thionyl halides followed by reaction with ammonia at high temperatures and pressures affords quantitative end-group conversion albeit with lower isolated yields of 60–73% [21,25]. Perhaps the most commonly employed approach is the three-step strategy involving conversion of the PEG hydroxyl end-groups to azido end-groups via the corresponding sulfonate or halide derivatives followed by reduction to amino end-groups with either triphenyl phosphine (PPh_3_) [26,29,33] or lithium aluminium hydride (LiAlH_4_) in anhydrous solvents [29,30], or hydrogenation over palladium on carbon (Pd/C) [32].

Despite several strategies being available for the conversion of hydroxyl-terminated PEG to amino-terminated PEG, these methods have several limitations. Firstly, these strategies have only been reported for the conversion of PEG with molecular weights <20 kDa, most likely due to the reduced solubility of higher molecular weight PEG in appropriate solvents [34]. Secondly, some of these strategies require highly flammable, corrosive and toxic reagents (e.g., hydrazine, LiAlH_4_, hydrogen), and specialised conditions (e.g., anhydrous conditions) and equipment (e.g., high pressure reactors), which can raise safety concerns and may not be readily available. Thirdly, reactions with PPh_3_ can result in contamination of the product with PPh_3_ and triphenylphosphine oxide (PPh_3_O) [35,36,37], which are progressively harder to remove as the molecular weight of the PEG increases and can lead to reduced yields. Finally, many of these methods have only been demonstrated for PEG and may not be applicable to the conversion of secondary alcohols, such as those found in poly(propylene glycol)s (PPGs) [38]. Therefore, the introduction of a practical, facile, safe and high-yielding strategy for the quantitative conversion of hydroxy-terminated polyglycols to amino-terminated polyglycols with a broad range of molecular weights and architectures would be of great utility and significance for laboratory research.

While the conversion of hydroxyl-terminated PEGs to their corresponding azides has been extensively reported with high isolated yields and quantitative end-group conversion [25,28], their conversion to amines can be more challenging. Therefore, we aimed to identify a novel strategy that would allow the efficient and safe reduction of azides to amines, as well as the facile isolation of the product without contamination. To achieve this, we investigated reducing conditions using inorganic reagents [39,40,41,42,43,44,45] that would allow rapid isolation of the amino-terminated PEGs via a simple solvent extraction, and could be extended to other polyglycols. Previously, Mi and co-workers reported the reduction of aromatic azides using zinc (Zn) in the presence of ammonium chloride (NH_4_Cl) with excellent isolated yields (~90%) [46]. Therefore, in this study we investigated the suitability of this approach for the reduction of azido-terminated polyglycols to their amino-terminated counterparts from their corresponding hydroxyl-terminated derivatives, and the suitability of this approach to a continuous reagent addition process without the need for intermediate isolation steps.

## 2. Materials and Methods

### 2.1. Materials

α,ω-Dihydroxy PEG (*M_n_* ~ 1.5 and 10 kDa), α-methoxy-ω-hydroxy PEG (*M_n_* ~ 5 kDa), α,ω-dihydroxy Pluronic^®^ F-127 (*M_n_* ~ 12.6 kDa), α-butoxy-ω-hydroxy PPG (*M_n_* ~ 340 Da), methanesulfonyl chloride (MsCl; ≥99.7%), zinc (Zn; granular, 20-30 mesh, ACS reagent, ≥99.8%), triethylamine (TEA; ≥99.5%), ammonium chloride (NH_4_Cl; ≥99.5%) and deuterated chloroform (CDCl_3_, 99.8 atom % D) were purchased from Sigma–Aldrich. Hydroxy-terminated 4-armed PEG (*M_n_* ~ 10 kDa) was purchased from creative PEG works. α,ω-Dihydroxy PEG (*M_n_* ~ 35 kDa) was purchased from Fluka. α,ω-Dihydroxy PEG (*M_n_* ~ 4 kDa) was purchased from Ajax. Sodium Azide (NaN_3_; ≥99%), dichloromethane (DCM), tetrahydrofuran (THF) and *N*,*N*-dimethylformamide (DMF) were all of analytical grade and were purchased from ChemSupply (Gillman, Australia). Methanol (MeOH) of HPLC grade was purchased from ChemSupply (Gillman, Australia). All reagents were used as received. Ultrapure water with a resistivity of >18.2 MΩ.cm was obtained from a Sartorius Arium Pro ultrapure water system (Göttingen, Germany). Ultrahigh purity argon (99.999%) was purchased from BOC (BOC Gas, Australia).

### 2.2. Methods

Proton nuclear magnetic resonance (^1^H NMR) spectrometry was conducted using a 500 MHz Bruker Avance Ⅱ NMR spectrometer (Billerica, MA, USA) operating at 500 MHz and 25 °C. Spectra were recorded in CDCl_3_ using the residual CHCl_3_ signal as an internal reference. Gel permeation chromatography (GPC) was performed on a Shimadzu Prominence liquid chromatography system (Kyoto, Japan) coupled with a Shimadzu differential refractive index (RID-20A, λ = 633 nm), using either THF (40 °C) or 1:1 *v*/*v* MeOH:water as the mobile phase (50 °C). For organic-based (THF) GPC, two Shimadzu columns (GPC8025D, GPC-80MD) were used in series with a flow rate of 1 mL/min. For aqueous-based (1:1 *v*/*v* MeOH:water) GPC, two Aglient columns (PL aquagel-OH MIXED-M (8 µm), PL aquagel-OH 20 (5 µm)) were used in series with a flow rate of 0.8 mL/min. Column calibration curves were obtained using narrow dispersity (Ð) PEG standards (Polymer Standards Service GmbH). MALDI ToF MS was performed on a Bruker Autoflex III Mass Spectrometer (Billerica, MA, USA) operating in positive/linear or reflection/positive mode. In general, the analyte, matrix and cationisation agent were dissolved in THF at concentrations of 10, 10 and 5 mg/mL, respectively, and then mixed in a ratio of 10:1:1. Subsequently, 0.2 μL of this solution was spotted onto a ground steel target plate and the solvent was allowed to evaporate prior to analysis. PEG standards of the appropriate molecular weight range were used as external calibrants. FlexAnalysis (Bruker, Billerica, MA, USA) and Polymerix (Sierra Analytics, Modesto, CA, USA) were used to analyse the data. Attenuated total reflectance Fourier transform infrared (ATR-FTIR) analysis was conducted on a Shimadzu IRSpirit (Kyoto, Japan) recording across the range of 400 to 3500 cm^−1^ at a resolution of 4 cm^−1^.

### 2.3. Procedures

#### 2.3.1. Synthesis of Mesylate-Terminated Polyglycols

Mesylate-terminated polyglycols were prepared according to published literature procedures [25,28] with modification. The hydroxyl-terminated polyglycol and TEA (4 mol equiv. per hydroxyl group) were dissolved with stirring in DCM (5 mL per 1 g of polymer) in a two-necked round-bottomed flask under a flow of Argon. The mixture was cooled in an ice bath, and methanesulfonyl chloride (4 mol equiv. per hydroxyl group) was added dropwise. The flask was sealed, the ice bath was removed and the mixture was stirred at ambient temperature for 16 h. The reaction mixture was transferred to a centrifuge tube along with ultrapure water (10 mL per 5 mL of DCM) and vortexed (1 min). The mixture was centrifuged (5000 rpm, 3 min) and the aqueous phase was separated and discarded. This process was repeated three times, and the organic phase was dried with MgSO_4_ and then filtered using a sintered funnel under vacuum. The filtrate was concentrated in vacuo and the residue was dried under high vacuum (0.1 mbar) to afford the desired mesylate-terminated polyglycols with isolated yields of 96–99%. Note, complete removal of the DCM is required to prevent reaction with sodium azide in subsequent steps and the formation of explosive derivatives [47,48].

#### 2.3.2. Synthesis of Azido-Terminated Polyglycols

Azido-terminated polyglycols were prepared according to the literature procedure of Ren et al. [49]. Briefly, mesylate-terminated polyglycol was dissolved in DMF (5 mL per 1 g of polymer) in a round-bottomed flask and NaN_3_ (5 mol equiv. per mesylate group) was added. The flask was stoppered, and the mixture was heated to 65 ℃ for 16 h. After cooling to ambient temperature, the mixture was filtered and the insoluble material was washed with ethanol (10 mL). The filtrate was concentrated in vacuo, and the resulting residue was dissolved in ultrapure water (5 mL per 1 g of polymer). The aqueous solution was then extracted with DCM (3 × 10 mL), and the combined organic extracts were dried (MgSO_4_) and filtered using a sintered funnel under vacuum. The filtrate was concentrated in vacuo, and the residue was dried under high vacuum (0.1 mbar) to afford the desired azido-terminated polyglycols with isolated yields of 77–98%.

#### 2.3.3. Synthesis of Amino-Terminated Polyglycols

Amine-terminated polyglycols were prepared by adapting and modifying the literature procedure reported by Lin et al. for the conversion of small molecule aromatic azides to amines [46]. Azido-terminated polyglycol was dissolved in THF (5 mL per 1 g polymer) in a round-bottomed flask, and ultrapure water (2 mL per 1 g polymer), NH_4_Cl (4 mol equiv. per azide group) and Zn (2 mol equiv. per azide group) were added sequentially. A water condenser was attached, and the mixture was refluxed for 72 h with stirring (note: the reaction time for synthesis of amino-terminated PEG_35k_ and PPG_0.3k_ was increased to 150 h). The reaction mixture was cooled to ambient temperature, diluted with 1 M NaOH (5 mL per 1 g polymer) and then extracted with DCM (5 × 10 mL). The combined organic extracts were dried (MgSO_4_) and filtered using a sintered funnel under vacuum. The filtrate was concentrated in vacuo, and the residue was dried under high vacuum (0.1 mbar) to afford the desired amino-terminated polyglycols with isolated yields of 82–99%.

#### 2.3.4. Sequential Reagent Addition Synthesis of α,ω-Diamino PEG_1.5k_

α,ω-Dihydroxy PEG_1.5k_ (0.5 g, 333 μmol) and TEA (0.116 mL, 833 μmol) were dissolved in THF (2 mL) with stirring in a 4 mL vial, which was fitted with a septa and purged with argon. Methanesulfonyl chloride (0.064 mL, 833 μmol) was added dropwise, and the mixture was stirred at ambient temperature for 16 h. The mixture was centrifuged (5000 rpm, 3 min) to remove the TEA hydrochloride salt, and NaN_3_ (0.21 g, 3.33 mmol) and DMF (2 mL) were added to the supernatant. The mixture was heated at 80 ℃ for 48 h with stirring and then cooled to ambient temperature. The mixture was filtered using a sintered funnel under vacuum, and the insolubles were washed with THF (3 mL). The filtrate was transferred to a vial, and ultrapure water (1 mL), NH_4_Cl (0.14 g, 2.67 mmol) and Zn (0.09 g, 1.33 mmol) were added sequentially. The mixture was heated to 80 ℃ for 72 h with stirring. After cooling to ambient temperature, 1 M NaOH (3 mL) was added, and the mixture was extracted with DCM (5 × 8 mL). The combined organic extracts were dried (MgSO_4_) and filtered using a sintered funnel under vacuum. The filtrate was concentrated in vacuo, and the residue was dried under high vacuum (0.1 mbar) to afford the desired amino-terminated PEG_1.5k_ with an isolated yield of 74%.

## 3. Results and Discussion

**Scheme 1:**
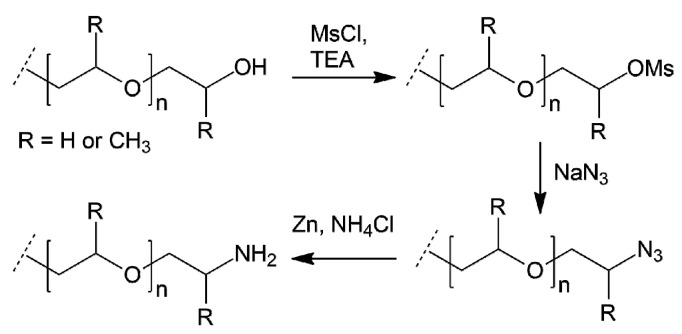
Synthetic scheme for the conversion of hydroxyl-terminated polyglycols to amino-terminated polyglycols via the corresponding mesylates and azides (R = H or CH_3_).

Amino end-group functionalised polyglycols were accessed from their corresponding hydroxy-terminated derivatives through a sequential three-step process consisting of mesylation [26,29] azidation [49] and reduction with Zn in the presence of NH_4_Cl (Scheme 1), without the need for anhydrous solvents or specialised equipment.

**Figure 1:**
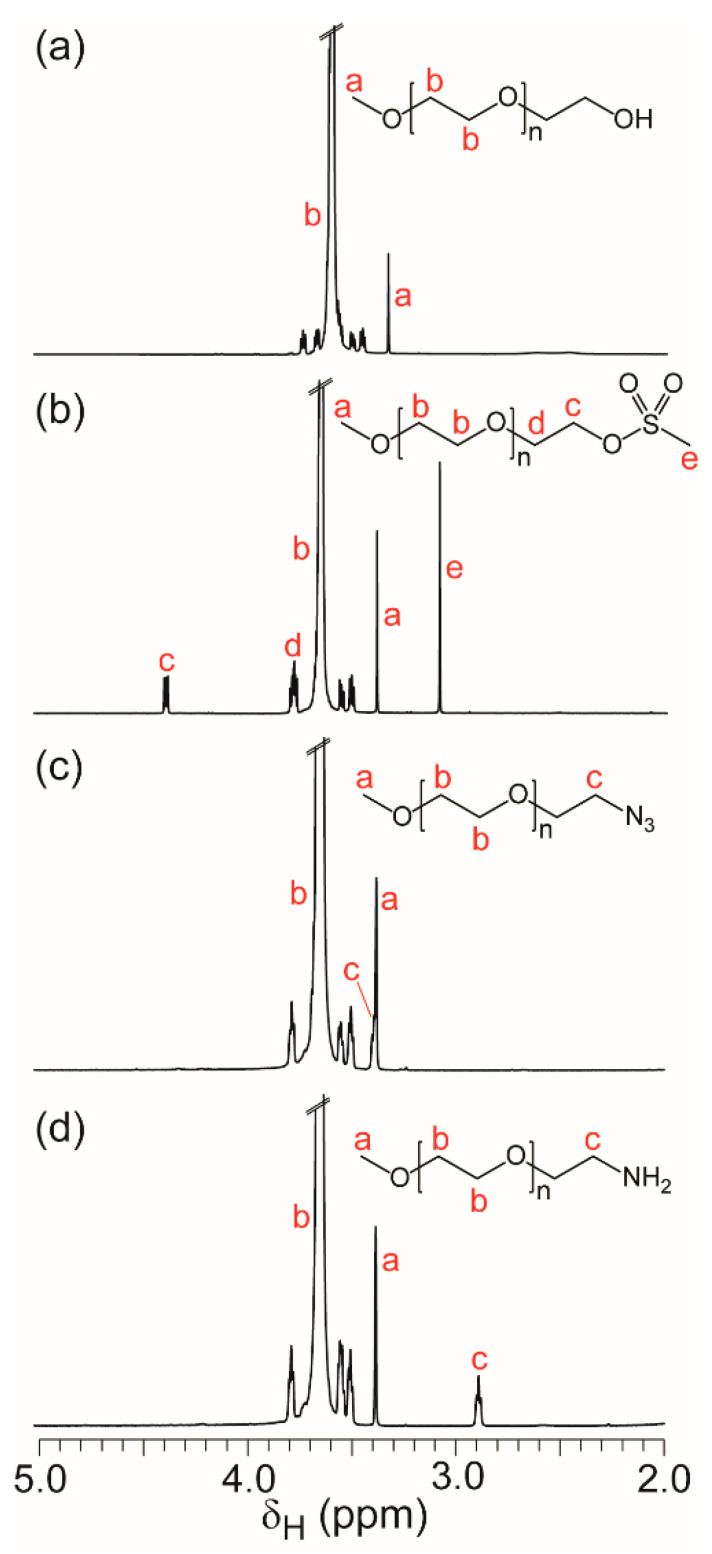
^1^H NMR spectra (500 MHz, 23 ℃, CDCl_3_) showing the conversion of the (**a**) α-methoxy-ω-hydroxy PEG_5k_ to its (**b**) α-methoxy-ω-mesylate, (**c**) α-methoxy-ω-azido and (**d**) α-methoxy-ω-amino derivatives.

To initially test this new approach, we used α-methoxy-ω-azido PEG_5k_ prepared via the traditional approach from its corresponding heterotelechelic α-methoxy-ω-hydroxy PEG_5k_. End-group transformations were followed by ^1^H NMR spectroscopic analysis and revealed the quantitative conversion of the ω-hydroxy PEG_5k_ to its mesylate and then azido derivatives (Figure 1a–c) as revealed by the appearance of characteristic resonances at δ_H_ 4.37 and 3.37 ppm for the terminal methylene protons adjacent to the mesylate and azido groups, respectively. Subsequently, reduction of the azido end-group to an amine with Zn was found to proceed highly efficiently, as revealed by the complete shift of the terminal methylene proton resonance from δ_H_ 3.37 to 2.88 ppm (Figure 1d). Comparison of the terminal α-methoxy (δ_H_ 3.38 ppm) and ω-amino methylene proton signal integrations indicated >99% conversion. The end-group conversion was further confirmed by ATR-FTIR, which revealed the absence of a vibration corresponding to the azide group in the α-methoxy-ω-amino PEG_5k_ (Figure S1) [50].

In addition, the simple solvent extraction work-up procedure allowed the α-methoxy-ω-amino PEG_5k_ to be isolated rapidly with a yield of 95% (>90% in three steps) without the presence of any impurities (Table 1), as confirmed by the absence of erroneous resonances in the NMR spectrum. In comparison, reduction of azido end-groups using the traditional Staudinger [26,29] reaction only resulted in a conversion of ~95%, and extensive purification via solvent extraction and precipitation (procedure in the Supplementary Information (SI)) failed to completely remove Ph_3_P and Ph_3_PO contaminates (Figure S2), resulting in a significant reduction in the isolated yield (~60%).

**Figure 2:**
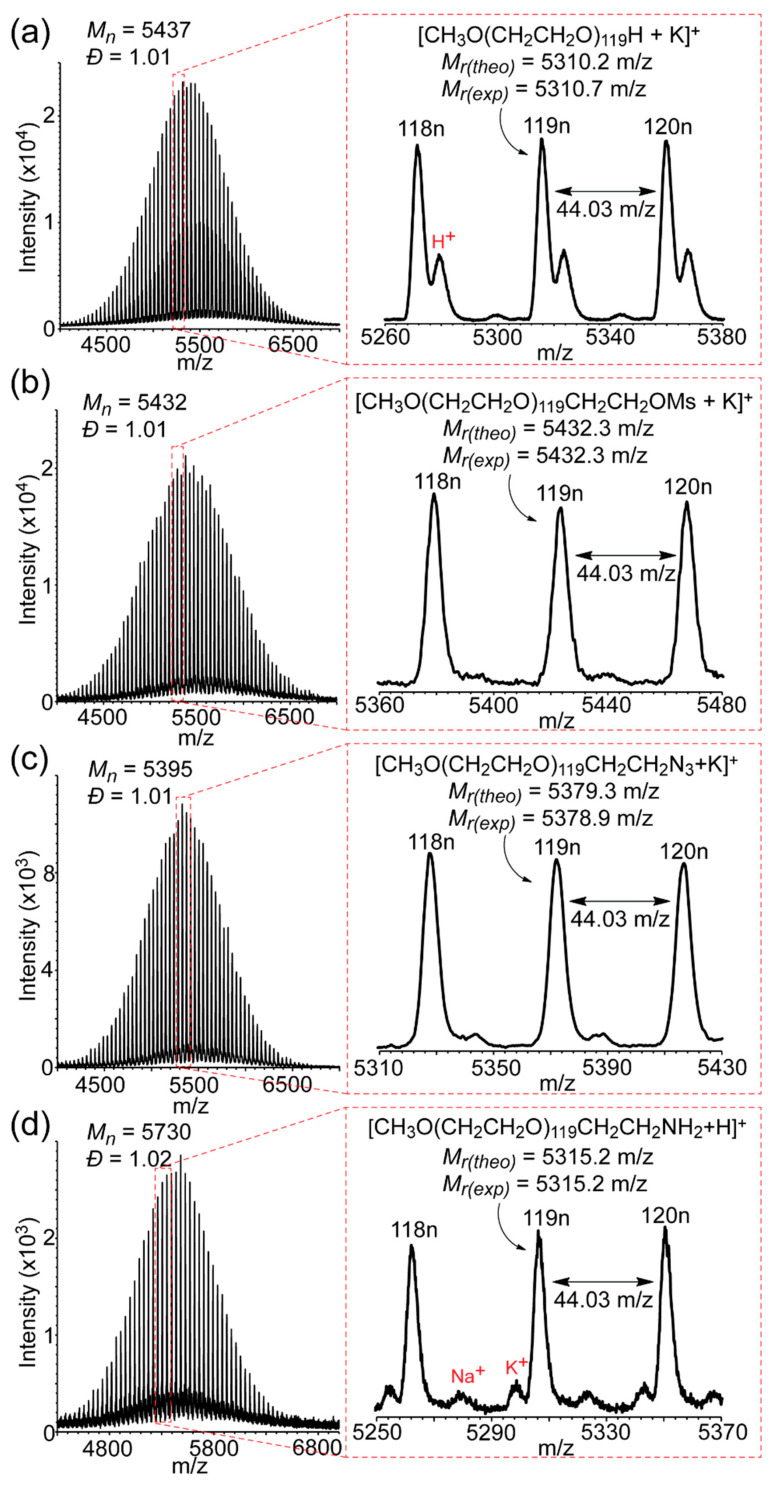
MALDI-ToF MS spectra showing the conversion of the (**a**) α-methoxy-ω-hydroxy PEG_5k_ to its (**b**) α-methoxy-ω-methylsulfonate, (**c**) α-methoxy-ω-azido and (**d**) α-methoxy-ω-amino derivatives; expansions show the 118–120 repeat unit region of the spectra along with the theoretical (*M_r_(theo)*) and experimentally (*M_r_(exp)*) determined average molecular mass values of polymers with n = 119.

To confirm the conversion of the end-groups, MALDI-ToF MS was conducted on the α-methoxy-ω-amino PEG_5k_ and each of its precursors, which revealed regular repeating series consist with PEG, and in all cases, average molecular mass (*M_r_*) values consistent with the potassium or proton adduct of the expected end-groups (Figure 2). For several of the PEG_5k_ derivatives, smaller series of peaks were also noted corresponding to other adducts. No series corresponding to precursor end-groups were noted for any of the transformations, confirming the complete conversion observed from NMR spectroscopy (Figure 1). Analysis of the MALDI-ToF MS results also provided molecular weight characteristics (number-average molecular weight (*M_n_*) and dispersity (Ð)) of the polymers, which were found to be consistent with the results obtained from NMR spectroscopy (Supplementary Materials, Table S1), as well as GPC, which revealed negligible change in the differential refractive index chromatograms throughout the functional group transformations (Figure S3).

Subsequently, we investigated the generalizability of the Zn reduction strategy to other azido-terminated polyglycols, including linear homotelechelic PEGs, star-shaped PEG, PPG and copolymers thereof (Pluronic^®^ F-127; PEG-PPG-PEG) (Table 1). In all cases, the amino-terminated polyglycols were prepared from the corresponding hydroxyl-terminated polyglycols using the three-step process previously described (vide supra), with the final step providing high isolated yields (82–99%). NMR spectroscopic analysis of the amino-terminated polyglycols and their precursors revealed complete transformation of the end-groups (>99% conversion) and the absence of impurities (Figures S4–S10), even for high-molecular-weight PEG_35k_. The conversion of α-butoxy-ω-hydroxyl PPG_0.3k_ to its corresponding α-butoxy-ω-amino PPG_0.3k_ also demonstrates that this approach is applicable to secondary groups [38]. From the NMR spectra, the *M_n_* values of the amino-terminated polyglycols were calculated (Table S1) and all in cases were found to be consistent with the *M_n_* values of the precursor PEG; for homotelechelic PEGs, the *M_n_* values of the amino-terminated PEGs were compared to those of the azido-terminated PEGs due to the absence of distinct end-group resonances from their hydroxyl-terminated derivatives.

For lower molecular weight polyglycols (PEG_1.5k_, PEG_4.0k_, PEG_10k_, 4-armed PEG_10k_ and PPG_0.3k_), conversion of the end-groups was further confirmed by MALDI-ToF MS, which revealed regular repeating series with monoisotopic mass (*M_mi_*) or *M_r_* values consistent with cation adducts of the expected end-groups (Figures S11–S15). Determination of the *M_n_* values of the polyglycol derivatives from each series via MALDI-ToF MS revealed negligible change as would be expected, and for most polymers the results were consistent with those obtained from NMR spectroscopy and GPC (Table S1 and Figures S16–S22). Notably, the GPC chromatograms of the α,ω-diamino PEG_10k_, α,ω-diamino PEG_35k_ and 4-armed amino-terminated PEG revealed the presence of slight shoulders or peaks to higher molecular weight, although it is unclear if these originate from physical aggregation or a side reaction that causes covalent coupling. Unfortunately, MALDI-ToF MS of the α,ω-diamino PEG_35k_ and PEG-PPG-PEG_13k_ polymers did not provide suitable peak resolution for further analysis due to their high molecular weight and combination of repeat units, respectively. Nevertheless, NMR spectroscopy and GPC revealed that the *M_n_* values of polymers in both series remained constant throughout the end-group transformations (Table S1).

As with any multi-step reaction sequence, it is often desirable to minimise time and effort associated with handling, processing and purification. Therefore, we investigated the end-group modification of α,ω-dihydroxy PEG_1.5k_ to α,ω-diamino PEG_1.5k_ via sequential reagent addition and without any intermediate isolation steps. The product was isolated with an overall yield of 74%, and ^1^H NMR spectroscopic analysis of the final product revealed >95% conversion of the end-groups to amines, with the remainder being unreduced azide groups (Figure S23). These end-groups were further confirmed by MALDI-ToF MS, which revealed a major repeating series consistent with the proton adduct of the α,ω-diamino PEG_1.5k_, and minor series consistent with sodium and potassium adducts (Figure S24). In addition, a minor series corresponding to α-azido-ω-amino PEG_1.5k_ was also detected, confirming previous observations in the NMR spectrum. As compared to many other synthetic strategies that rely on multiple reactions with incompatible reagents [26,29,30,31,32], the Zn reduction approach allows for the direct conversion of hydroxyl-terminated PEGs to their amino-terminated derivatives. An alternative one-step catalytic amination approach has also been reported by Abdollahi et al., using aqueous ammonia at high temperatures in sealed vessels [38]. While this approach provided an end-group conversion of 88% for relatively low-molecular-weight PEG (400 Da), the conversion efficiency decreased with increasing molecular weight and was found to be incompatible with PPG due to poor solubility.

## 4. Conclusions

Herein, we have demonstrated that the reduction of azido-terminated polyglycols with Zn is a facile and versatile approach for the synthesis of amino-terminated polyglycols with quantitative end-group transformation and high isolated yields (82–99%) owing to a simple solvent extraction approach that prevents the need for polymer precipitation to separate by-products. The Zn reduction strategy can be readily applied to polyglycols of different molecular weights and architectures, including high-molecular-weight polymers that can be challenging with other methods due to solvent compatibilities. Furthermore, this approach can also be used to efficiently convert secondary azides to amines, as demonstrated with PPG. Overall, the Zn reduction strategy is an attractive approach for the high-yielding preparation of high purity amino-terminated polyglycols that avoids the use of hazardous chemicals, specialised conditions and equipment, and lengthy work-up and isolation procedures. This new strategy provides a simple pathway to scale-up the preparation of amine-terminated polyglycols and generate amino-terminated PPG, opening up new possibilities for custom design in the research laboratory.

## Figures and Tables

**Table 1:** Isolated yield and end-group conversion of the functionalized polyglycols.

Polymer	Isolated Yield (%)				End-Group Conversion (%) ^a^
	Mesylate	Azide	Amine	Overall	
MeOPEG_5k_	96.9	98.6	94.4	90.3	>99
PEG_1.5k_	99.9	77.4	99.3	76.8	>99
PEG_4.0k_	99.9	95.3	94.5	90.0	>99
PEG_10k_	99.6	92.2	81.9	75.3	>99
PEG_35k_	98.9	84.8	88.9	74.6	>99
4-Armed PEG_10k_	98.6	96.8	93.1	88.8	>99
PEG-PPG-PEG_13k_	98.1	96.1	84.6	79.8	>99
*^n^*BuOPPG_0.3k_	96.6	98.6	92.8	88.4	>99

^a^ Conversion of hydroxyl to amine end-group determined from ^1^H NMR spectroscopy.

## Data Availability

The data presented in this study are available on request from the corresponding author.

## Supplementary Materials

The following are available online at https://www.mdpi.com/article/10.3390/polym13091403/s1, Figure S1: FTIR spectra of α-methoxy PEG_5k_ derivatives; Figure S2: NMR spectra of amino-terminated PEG_1.5k_ prepared via the Staudinger reaction; Figure S3: GPC chromatograms of α-methoxy PEG_5k_ derivatives; Figures S4–S10: NMR spectra showing the conversion of hydroxy- to amino-terminated polyglycols; Figures S11–S15: MALDI-ToF mass spectra showing the conversion of hydroxy- to amino-terminated polyglycols; Figures S16–S22: GPC chromatograms showing the conversion of hydroxy- to amino-terminated polyglycols; Figures S23–S24: GPC chromatograms and MALDI-ToF mass spectra for the sequential reagent addition synthesis. Table S1: Molecular weight characteristics of the polyglycols determined via ^1^H NMR spectroscopy, MALDI-ToF MS and GPC.
