# Supplementary material for: A Facile Strategy for the High Yielding, Quantitative Conversion of Polyglycol End-Groups to Amines
Source: Polymers (Basel). 2021 Apr 26;13(9):1403. doi: 10.3390/polym13091403 (PMC8123656; doi:10.3390/polym13091403)
Supplement: Supplementary file 1 [file polymers-13-01403-s001.zip › polymers-1198970-supplementary.pdf]

Supplementary Materials

A facile strategy for the high yielding, quantitative conversion of polyglycol end-groups to amines

Jie Yan, Paula Facal Marina and Anton Blencowe*

Applied Chemistry and Translational Biomaterials (ACTB) Group, Clinical and Health Sciences, University of South Australia, Adelaide, SA 5001, Australia; jie.yan@mymail.unisa.edu.au (J.Y.); paula.facalmarina@unisa.edu.au (P.F.M.)

* Correspondence: anton.blencowe@unisa.edu.au

Supporting Information

1. **Synthesis of α,ω-diamino $PEG_{1.5k}$ via the Staudinger reaction**
2. **Supporting Figures**
    a. **FTIR spectra of α-methoxy PEG_{5k} derivatives**
    b. **NMR spectra of $PEG_{1.5k}$ amine prepared from Staudinger reaction**
    c. **GPC chromatograms of methoxy PEG_{5k} and its derivatives**
    d. **NMR spectra**
    e. **MALDI-ToF mass spectra**
    f. **GPC chromatograms**
    g. **Figures for sequential reagent addition synthesis**
3. **Supporting Table**

References

1. Synthesis of α,ω-diamino $PEG_{1.5k}$ via the Staudinger reaction

The reduction of α,ω-diazido $PEG_{1.5k}$ to α,ω-diamino $PEG_{1.5k}$ via the Staudinger reaction was conducted according to published literature procedures [1, 2] with modification. α,ω-Diazido $PEG_{1.5k}$ (1.00 g, 667 μmol) and PPh_3 (1.40 g, 5.33 mmol, 4 equiv. per azide group) were dissolved in a 1:3 v/v ultrapure water:THF solution (8 mL) and stirred at 25 °C for 24 h. The reaction mixture was filtered with a sintered funnel under vacuum and the filtrate was concentrated *in vacuo* to remove the THF. 1 M HCl was added to the residue to adjust the pH to 1 and the resulting precipitate was filtered off using a sintered funnel under vacuum. The aqueous filtrate was then extracted with diethyl ether (3 × 10 mL), and the pH was adjusted to 13 using 1 M NaOH prior to extraction with DCM (3 × 5 mL). The combined DCM extracts were concentrated *in vacuo* to ~ 3 mL in volume and then precipitated into cold (0 ℃) diethyl ether (40 mL) with stirring. The precipitated polymer was collected via centrifugation (6000 rpm, 3 min), redissolved in DCM (3 mL) and precipitated into cold (0 ℃) diethyl ether (40 mL) with stirring; this step was repeated twice. The precipitated polymer was collected via centrifugation (5000 rpm, 3 min) and dried under high vacuum (0.1 mbar) to afford the product (0.59 g) contaminated with PPh_3 and PPh_3O.

2. Supporting Figures

a. FTIR spectra of α-methoxy PEG_{5k} derivatives

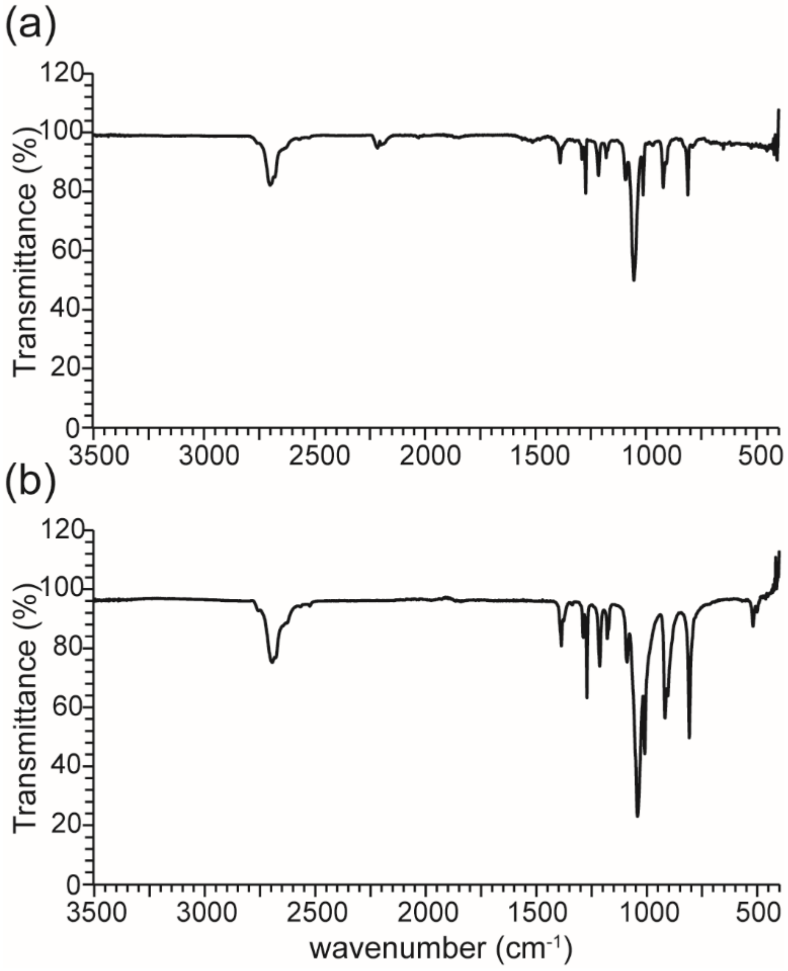

Figure S1. FTIR spectra of **(a)** α-methoxy-ω-hydroxy and **(b)** α-methoxy-ω-amino PEG_{5k}

b. NMR spectra of amino-terminated $PEG_{1.5k}$ prepared via the Staudinger reaction

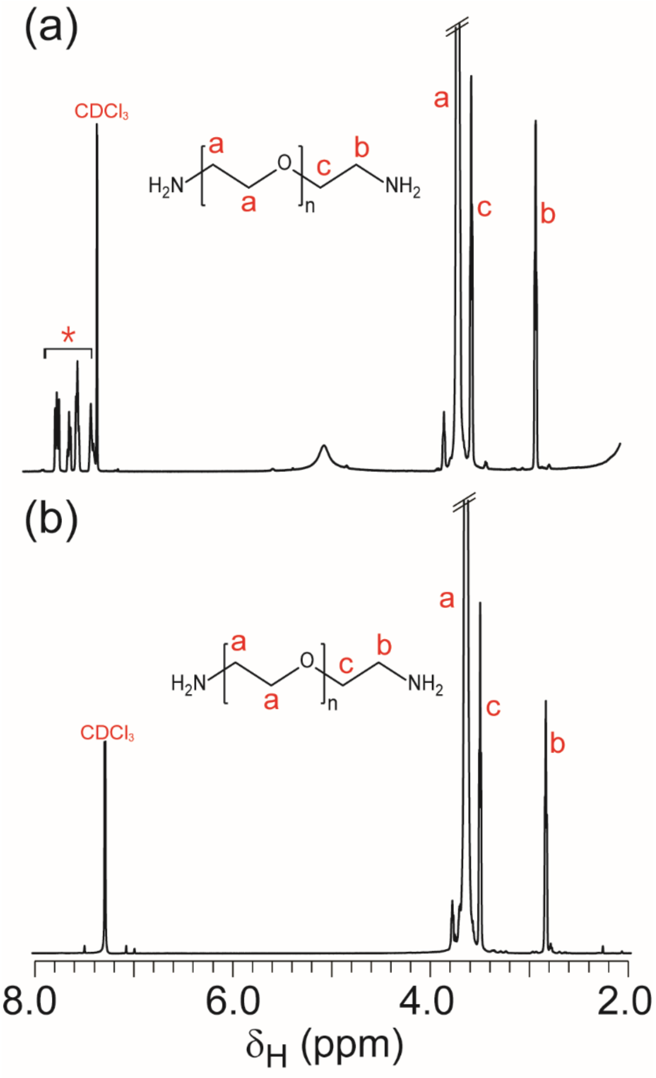

Figure S2. ^{1}H NMR spectra (500 MHz, 23 ℃, $CDCl_3$) of α,ω-diamine $PEG_{1.5K}$ prepared from α,ω-diazido $PEG_{1.5K}$ via **(a)** the Staudinger reaction (* indicates Ph_3P and Ph_3PO impurities present after purification) and **(b)** Zn / NH_4Cl reduction.

c. GPC chromatograms of α-methoxy PEG_{5k} derivatives

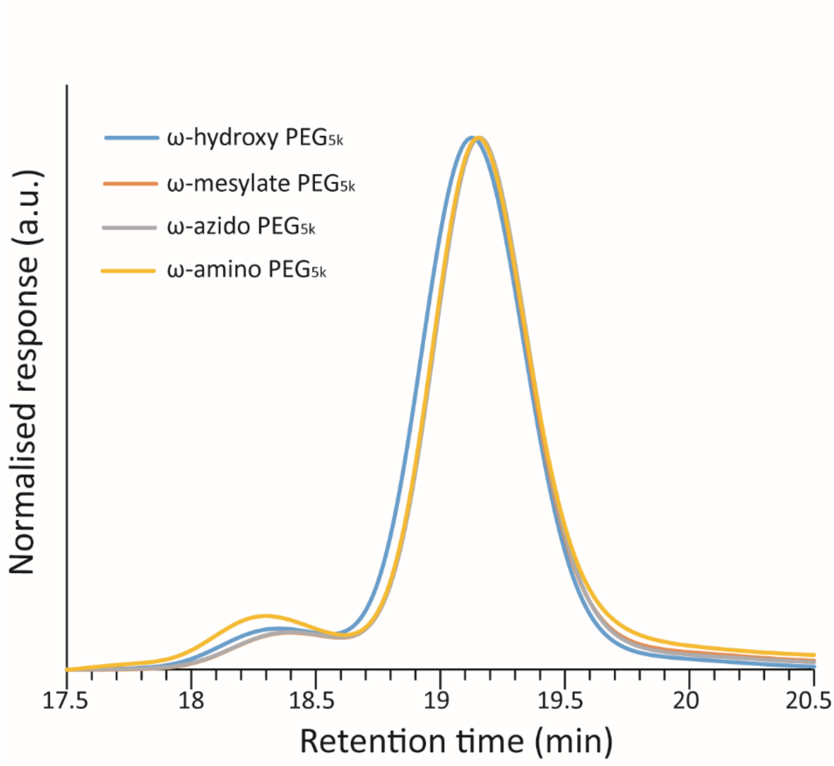

Figure S3. GPC chromatograms of the α-methoxy-ω-hydroxy, α-methoxy-ω-mesylate, α-methoxy-ω-azido and α-methoxy-ω-amino PEG_{5k} derivatives recorded using THF as the mobile phase.

d. NMR spectra showing the conversion of hydroxy- to amino-terminated polyglycols

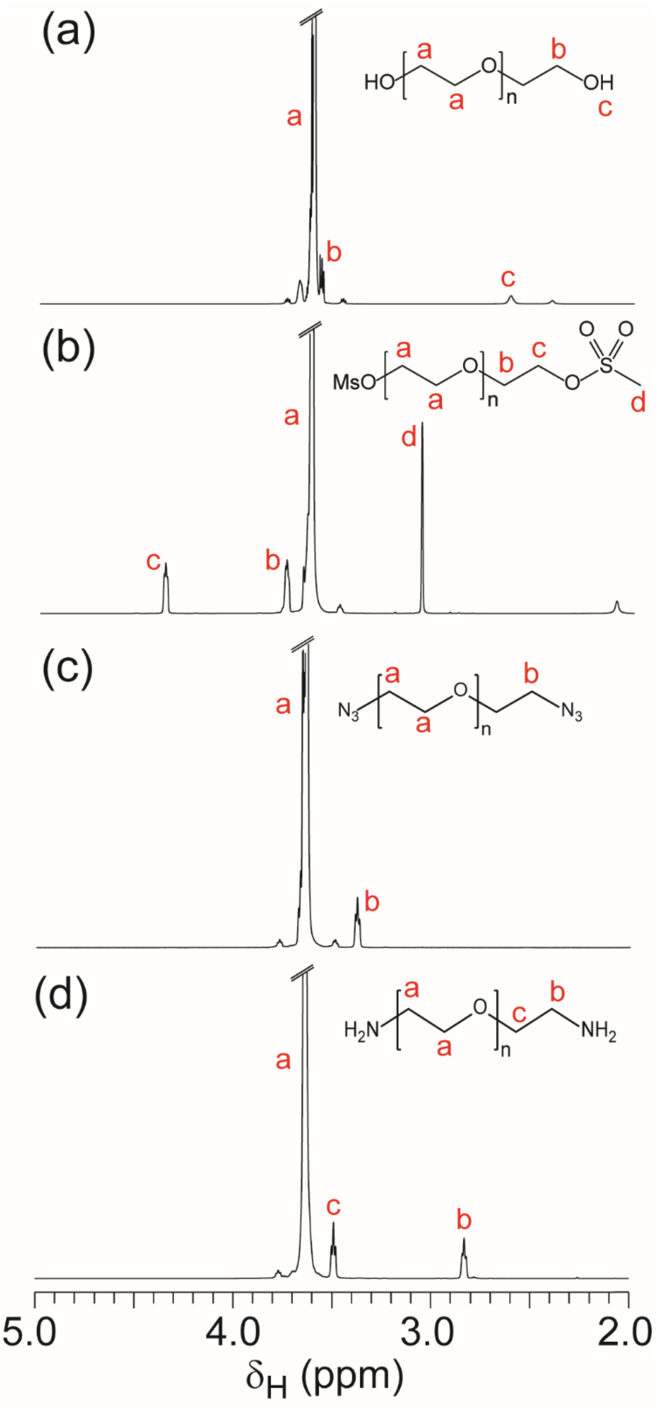

Figure S4. ^{1}H NMR spectra (500 MHz, 23 ℃, $CDCl_3$) showing the conversion of the **(a)** α,ω-dihydroxy $PEG_{1.5k}$ to its **(b)** α,ω-dimesylate, **(c)** α,ω-diazido and **(d)** α,ω-diamino $PEG_{1.5k}$ derivatives.

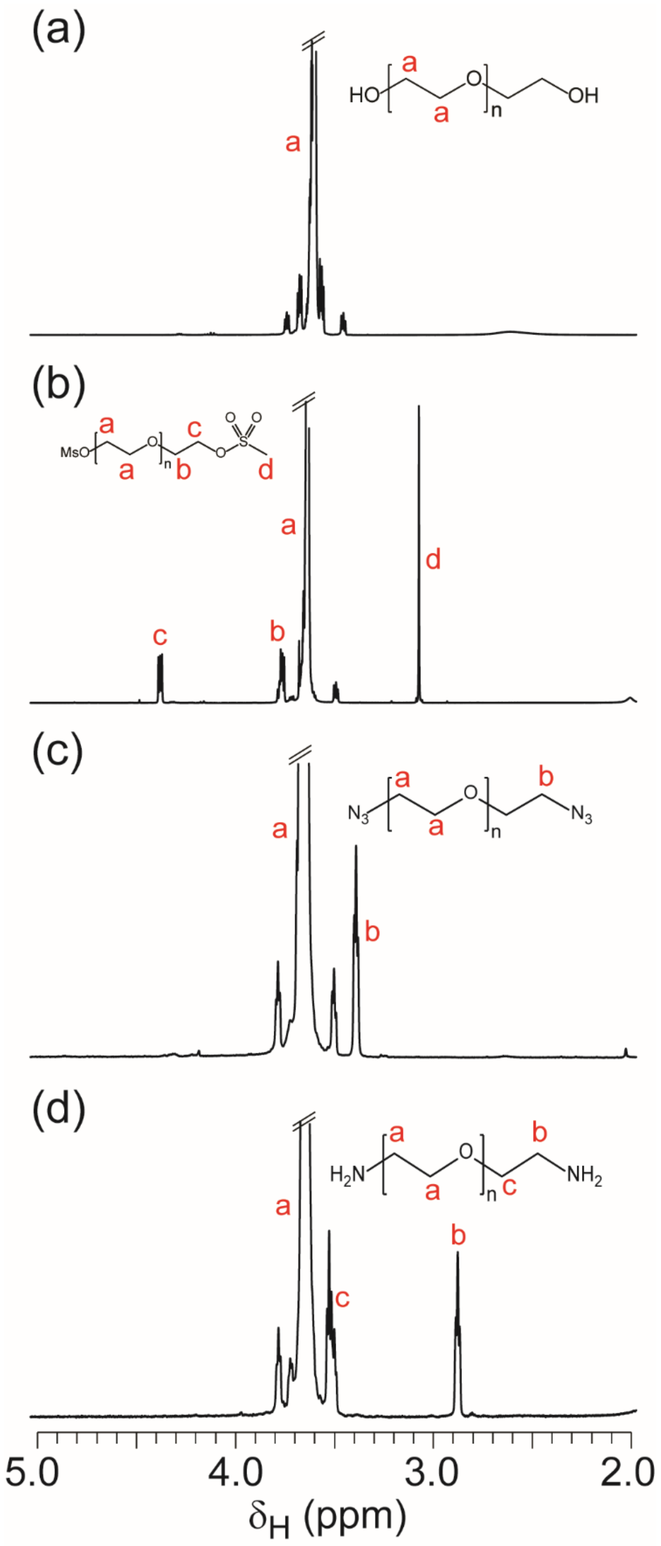

Figure S5. ^{1}H NMR spectra (500 MHz, 23 ℃, $CDCl_3$) showing the conversion of the **(a)** α,ω-dihydroxy PEG_{4k} to its **(b)** α,ω-dimesylate, **(c)** α,ω-diazido and **(d)** α,ω-diamino PEG_{4k} derivatives.

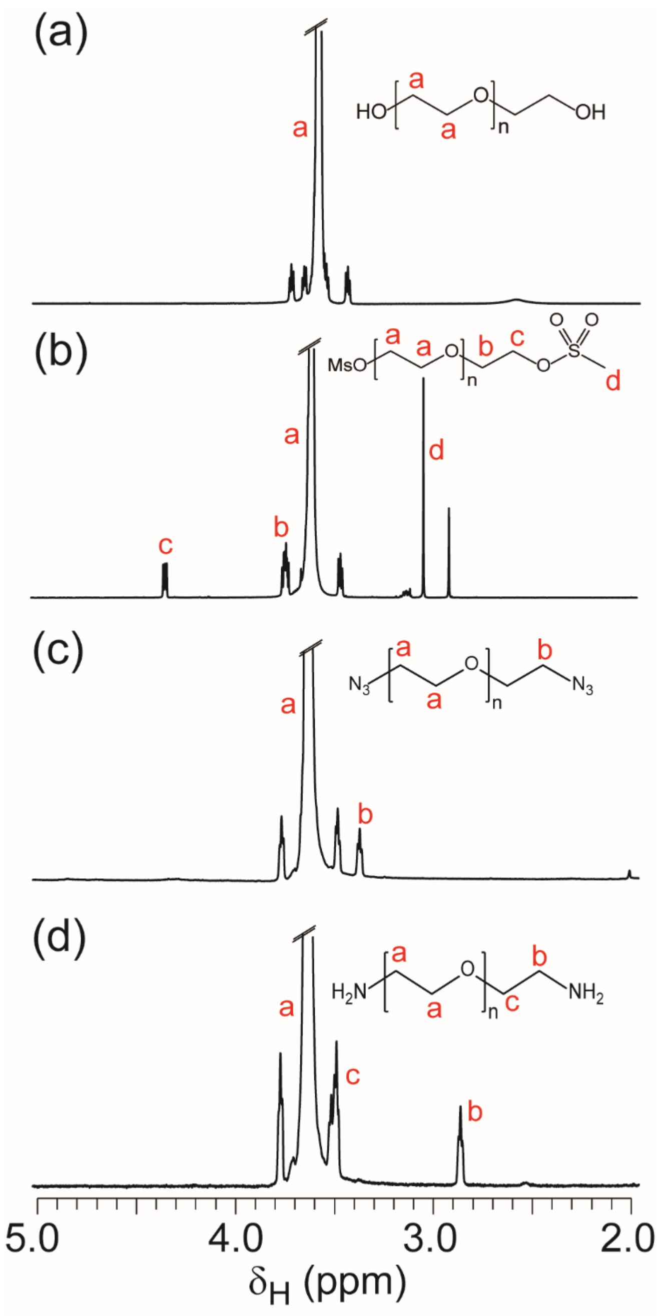

Figure S6. ^{1}H NMR spectra (500 MHz, 23 ℃, $CDCl_3$) showing the conversion of the **(a)** α,ω-dihydroxy PEG_{10k} to its **(b)** α,ω-dimesylate, **(c)** α,ω-diazido and **(d)** α,ω-diamino PEG_{10k} derivatives.

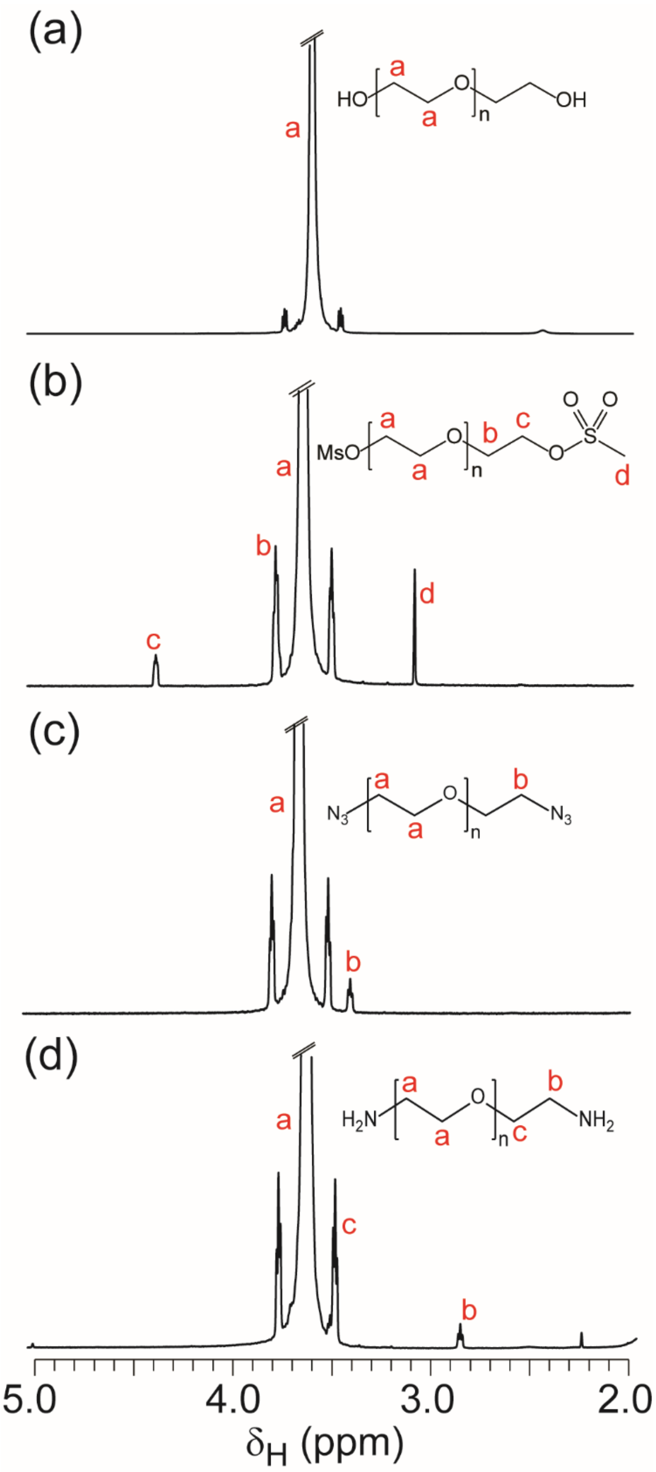

Figure S7. ^{1}H NMR spectra (500 MHz, 23 ℃, $CDCl_3$) showing the conversion of the **(a)** α,ω-dihydroxy PEG_{35k} to its **(b)** α,ω-dimesylate, **(c)** α,ω-diazido and **(d)** α,ω-diamino PEG_{35k} derivatives.

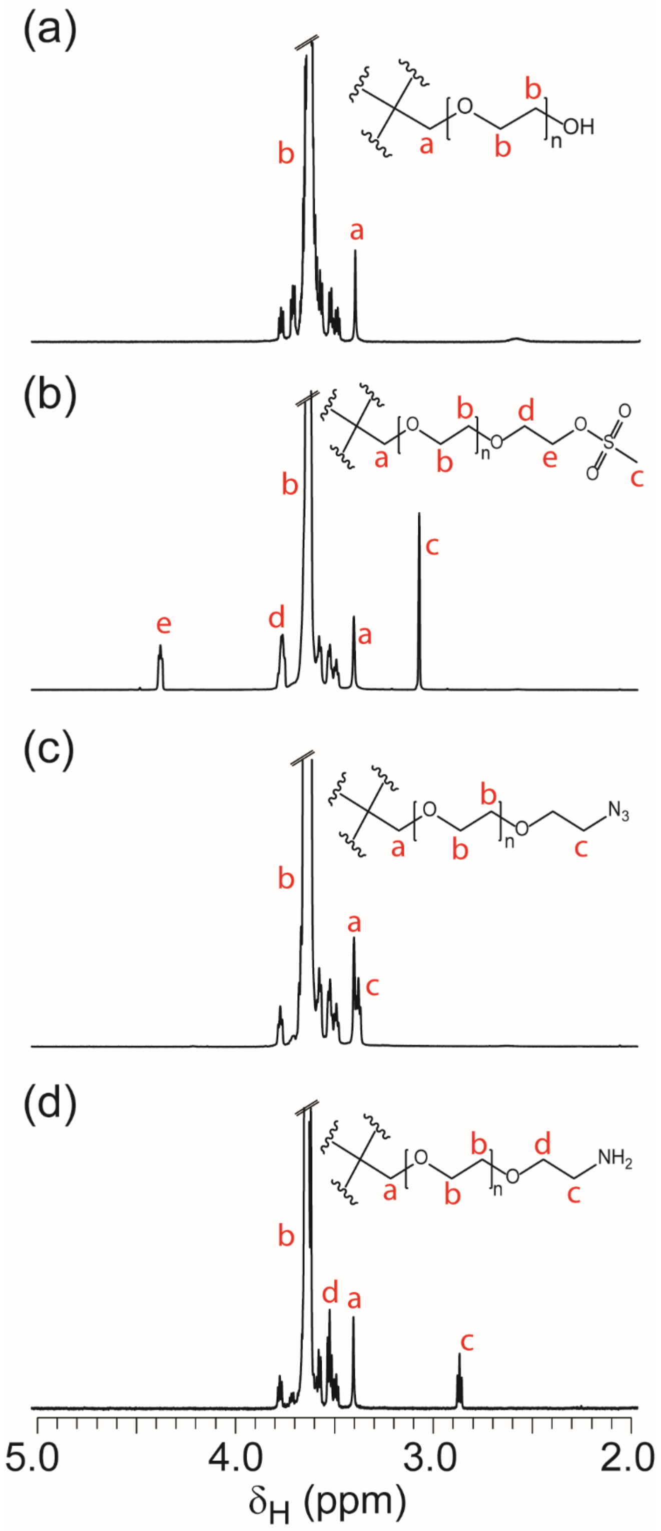

Figure S8. ^{1}H NMR spectra (500 MHz, 23 ℃, $CDCl_3$) showing the conversion of the **(a)** 4-armed tetrahydroxy PEG_{10k} to its **(b)** mesylate-, **(c)** azido-, and **(d)** amino-terminated derivatives.

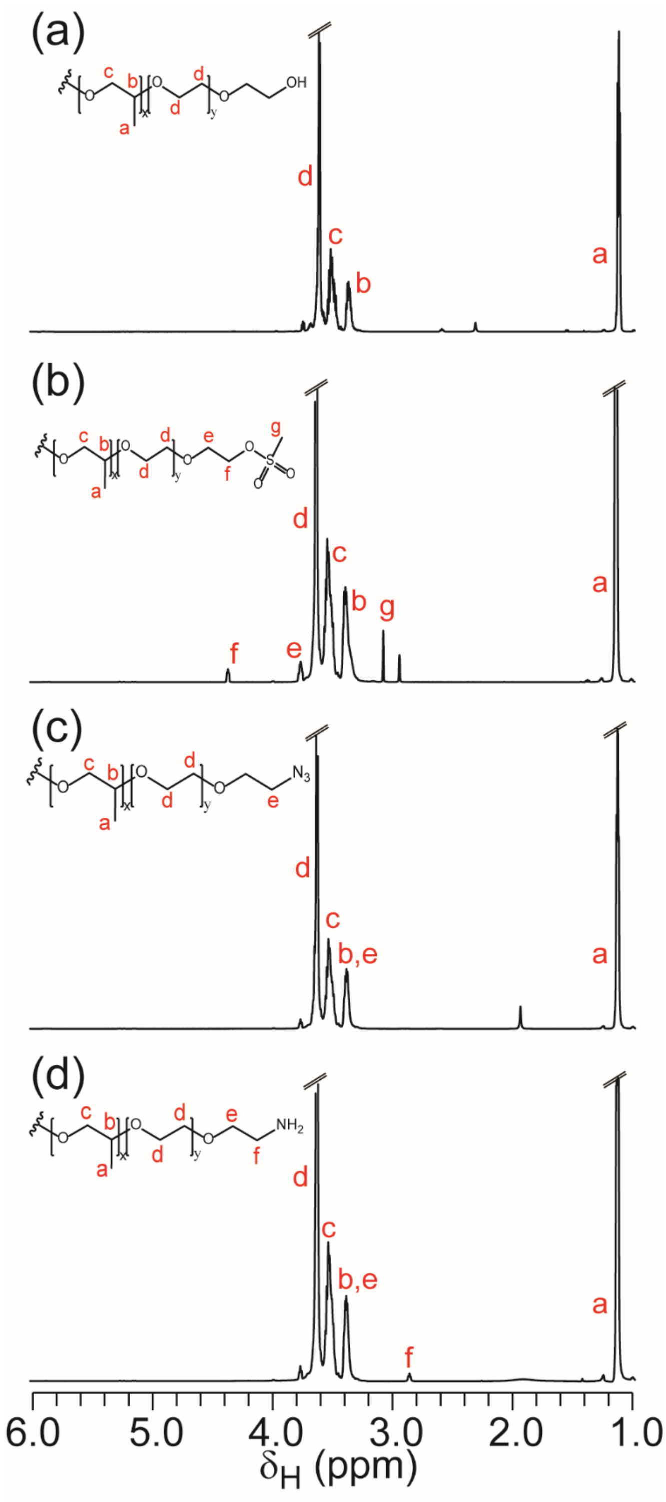

Figure S9. ^{1}H NMR spectra (500 MHz, 23 ℃, $CDCl_3$) showing the conversion of **(a)** Pluronic® F-127 (α,ω-dihydroxy PEG-PPG-PEG triblock copolymer, M_n ~ 12600) to its **(b)** α,ω-dimesylate, **(c)** α,ω-diazido and **(d)** α,ω-diamino derivatives.

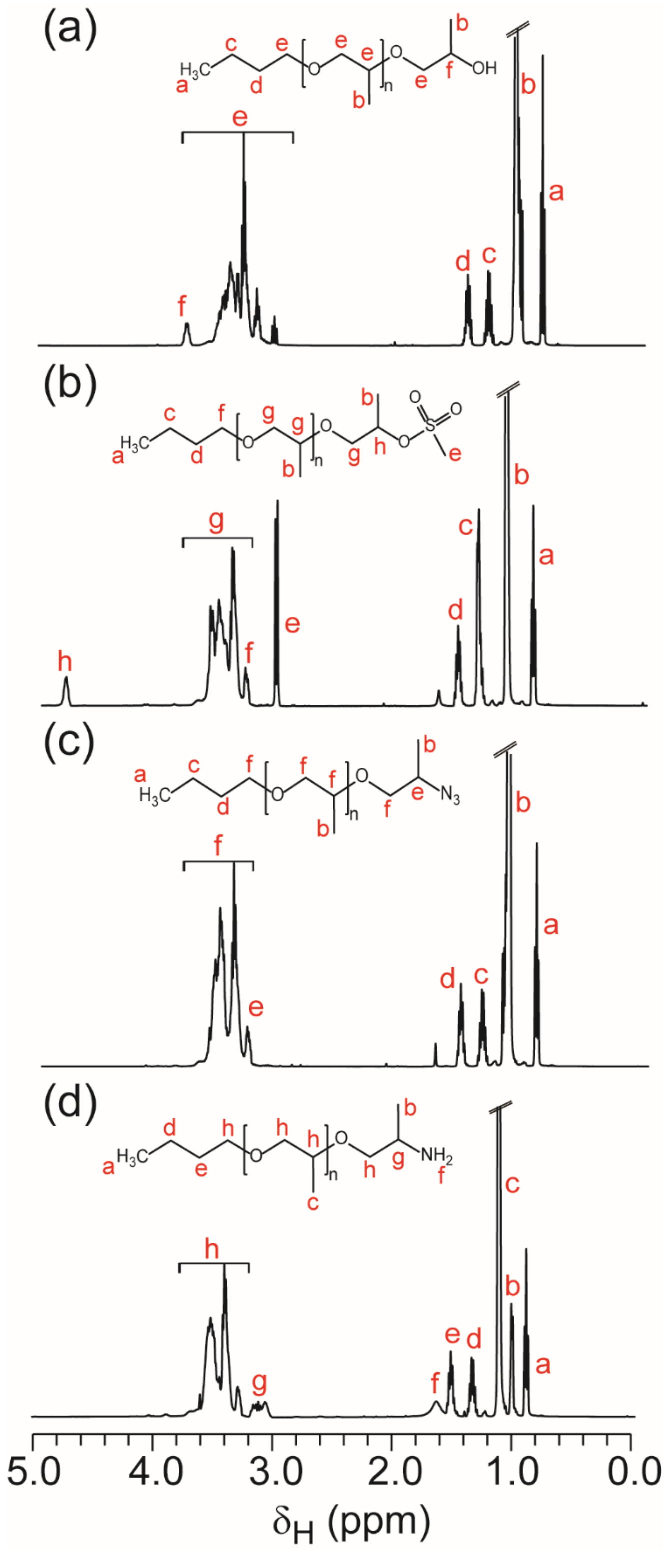

Figure S10. ^{1}H NMR spectra (500 MHz, 23 ℃, $CDCl_3$) showing the conversion of **(a)** α-butyl-ω-hydroxy PPG to its **(b)** α-butyl-ω-mesylate, **(c)** α-butyl-ω-azido and **(d)** α-butyl-ω-amino PPG derivatives.

e. MALDI-ToF mass spectra showing the conversion of hydroxy- to amino-terminated polyglycols

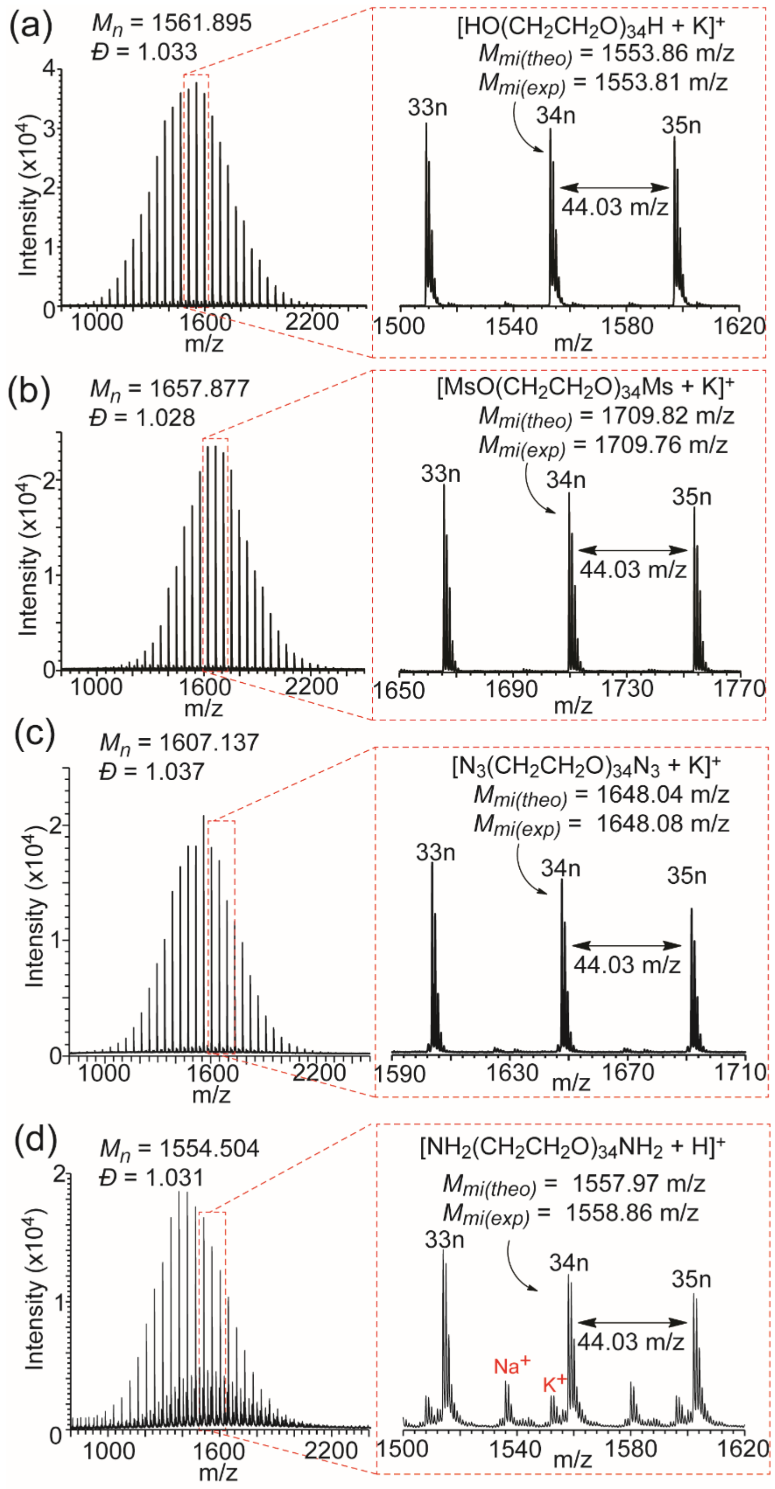

Figure S111. MALDI-ToF mass spectra showing the conversion of the **(a)** α,ω-dihydroxy PEG$_{1.5k}$ to its **(b)** α,ω-dimesylate, **(c)** α,ω-diazido and **(d)** α,ω-diamine PEG$_{1.5k}$ derivatives: expansions show 33-35 repeat unit region of the spectra with the theoretical ($M_{mi(theo)}$) and experimentally determined ($M_{mi(exp)}$) monoisotopic mass values for the potassium or proton adducts of polymers with n = 34.

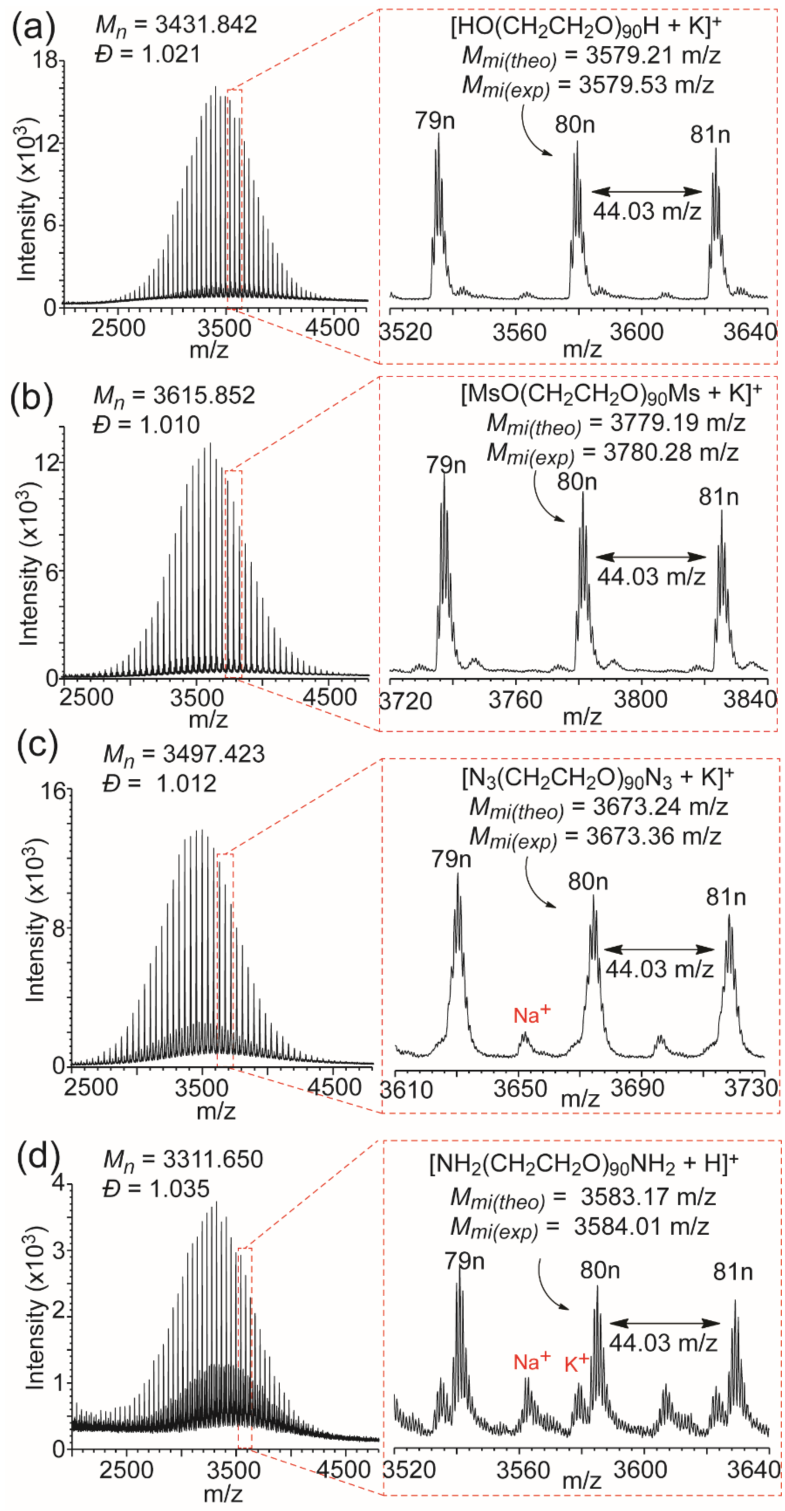

Figure S12. MALDI-ToF mass spectra showing the conversion of the **(a)** α,ω-dihydroxy PEG4k to its **(b)** α,ω-dimesylate, **(c)** α,ω-diazido and **(d)** α,ω-diamine PEG4k derivatives: expansions show 79-81 repeat unit region of the spectra with the theoretical ($M_{mi(theo)}$) and experimentally determined ($M_{mi(exp)}$) monoisotopic mass values for the potassium or proton adducts of polymers with n = 80.

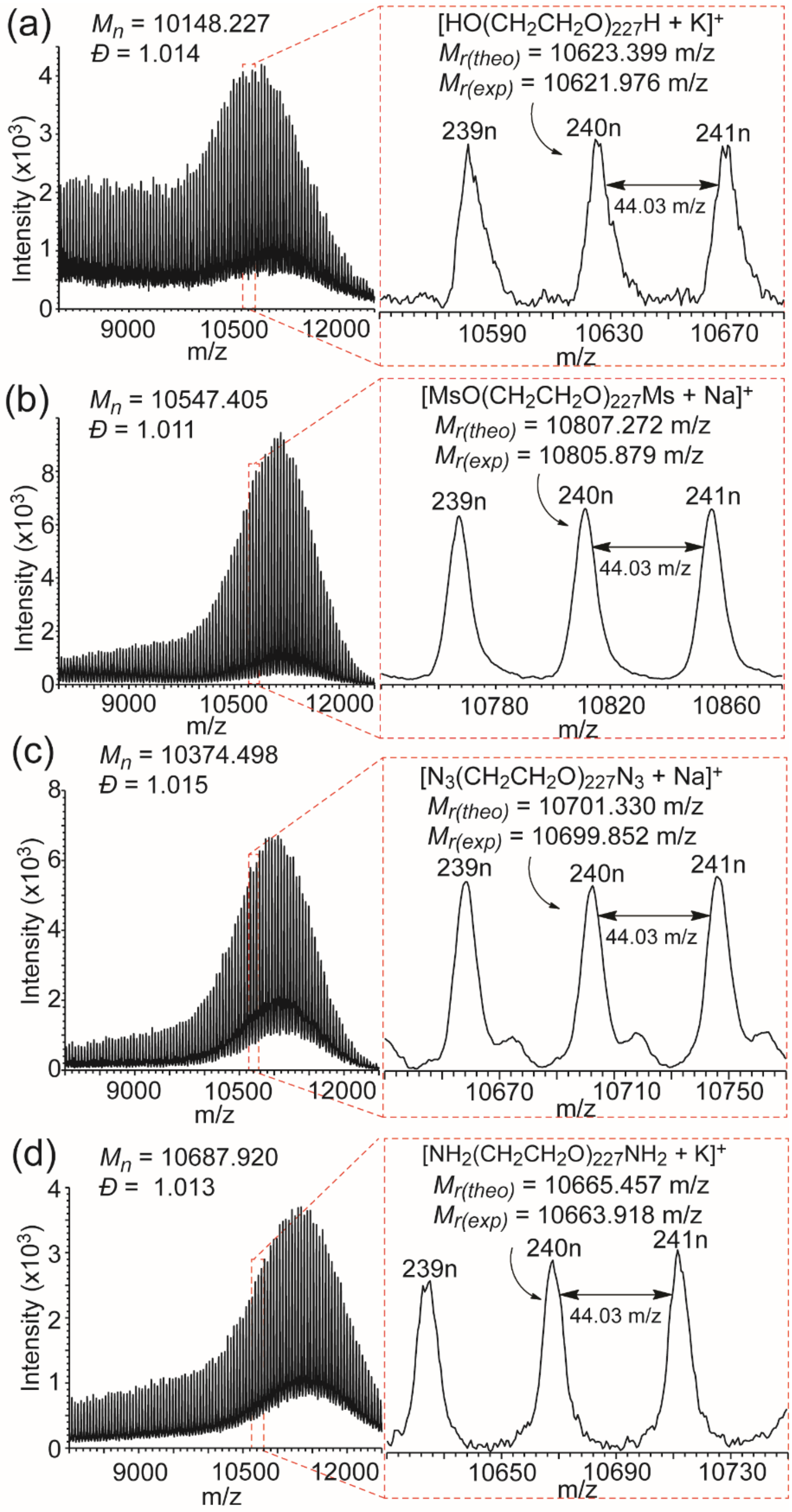

Figure S13. MALDI-ToF mass spectra showing the conversion of the **(a)** α,ω-dihydroxy PEG_{10k} to its **(b)** α,ω-dimesylate, **(c)** α,ω-diazido and **(d)** α,ω-diamine PEG_{10k} derivatives: expansions show 239-241 repeat unit region of the spectra with the theoretical ($M_{r(theo)}$) and experimentally determined ($M_{r(exp)}$) average molecular mass values for polymers with n = 240.

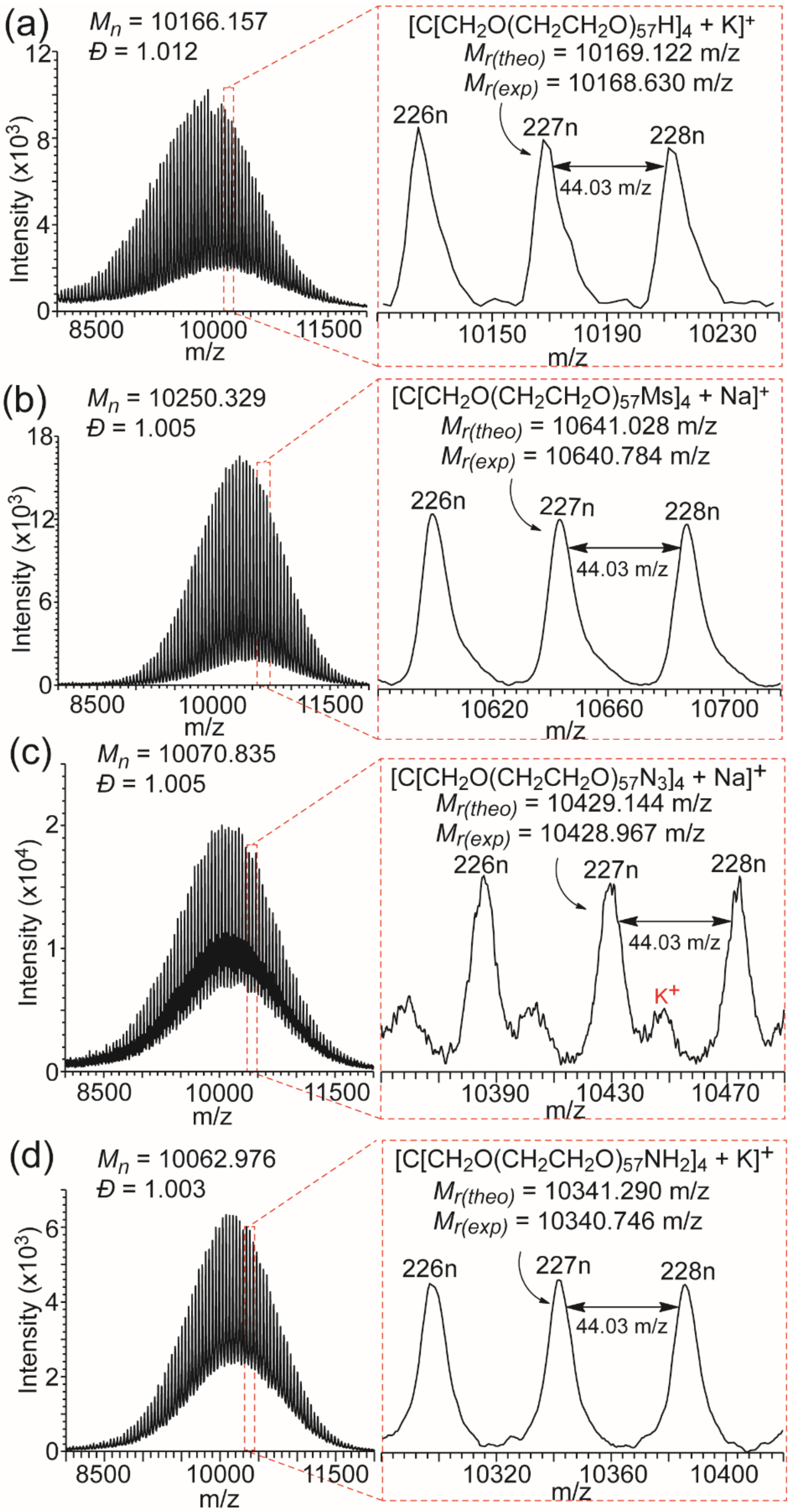

Figure S14. MALDI-ToF mass spectra showing the conversion of the **(a)** 4-armed tetrahydroxy PEG_{10k} to its **(b)** mesylate-, **(c)** azido-, and **(d)** amino-terminated derivatives: expansions show 226-228 repeat unit region of the spectra with the theoretical ($M_{r(theo)}$) and experimentally determined ($M_{r(exp)}$) average molecular mass values for polymers with n = 227.

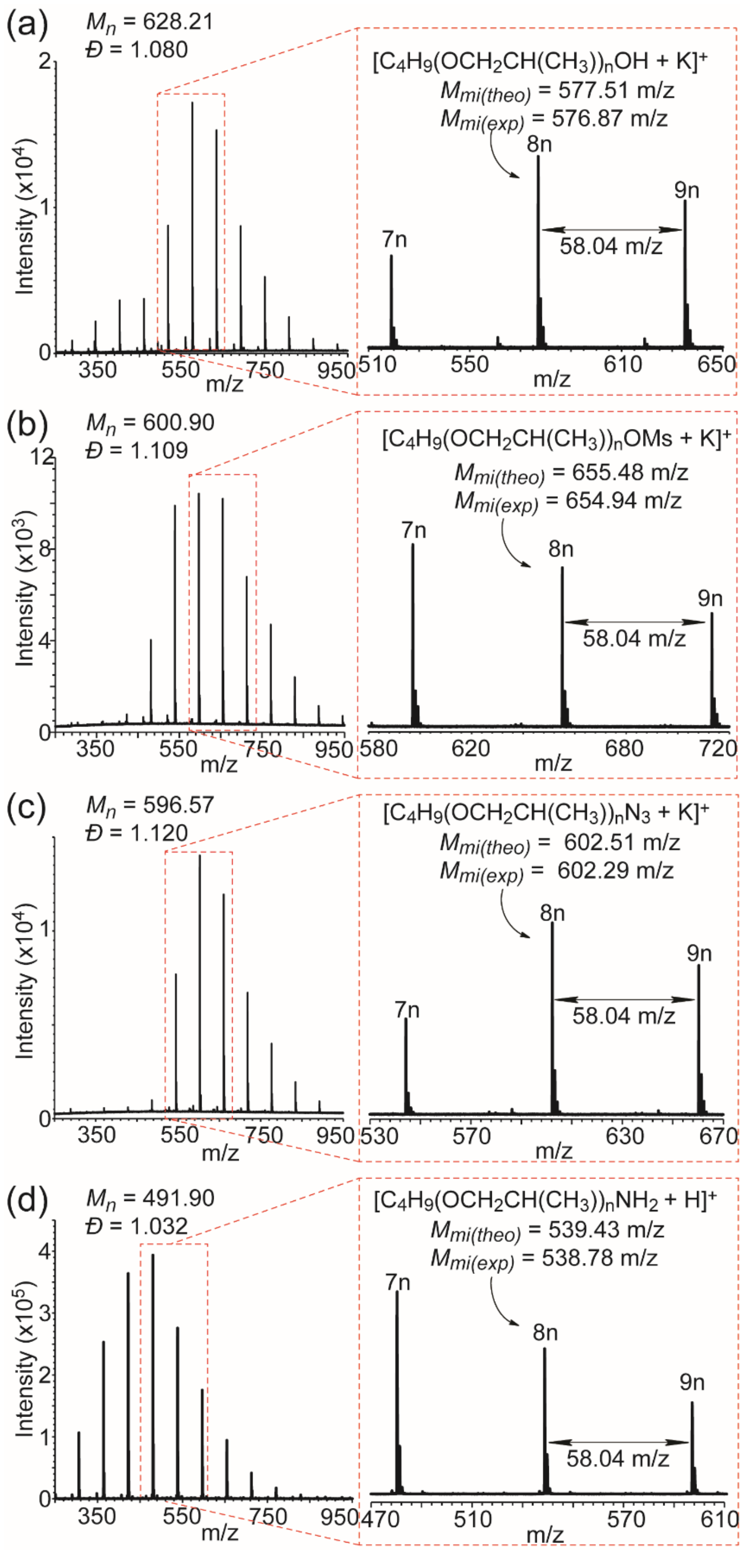

Figure S15. MALDI-ToF mass spectra showing the conversion of **(a)** α-butyl-ω-hydroxy PPG to its **(b)** α-butyl-ω-mesylate, **(c)** α-butyl-ω-azido and **(d)** α-butyl-ω-amino PPG derivatives: expansions show 7-9 repeat unit region of the spectra with the theoretical ($M_{mi(theo)}$) and experimentally determined ($M_{mi(exp)}$) monoisotopic mass values for the potassium or proton adducts of polymers with n = 8.

f. GPC chromatograms showing the conversion of hydroxy- to amino- terminated polyglycols

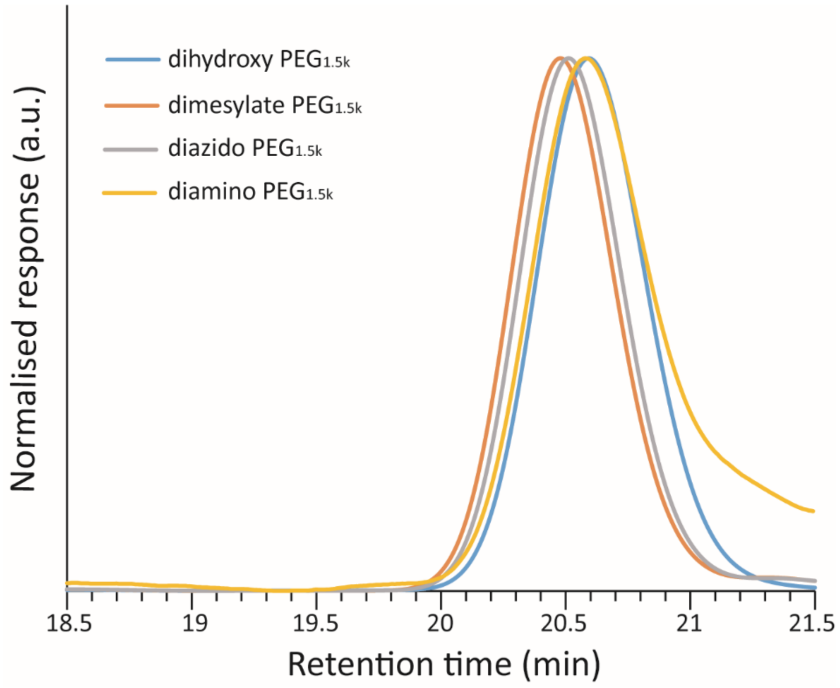

Figure S16. GPC chromatograms showing the conversion of the α,ω-dihydroxy $PEG_{1.5k}$ to its α,ω-dimesylate, α,ω-diazido and α,ω-diamino $PEG_{1.5k}$ derivatives recorded using THF as the mobile phase.

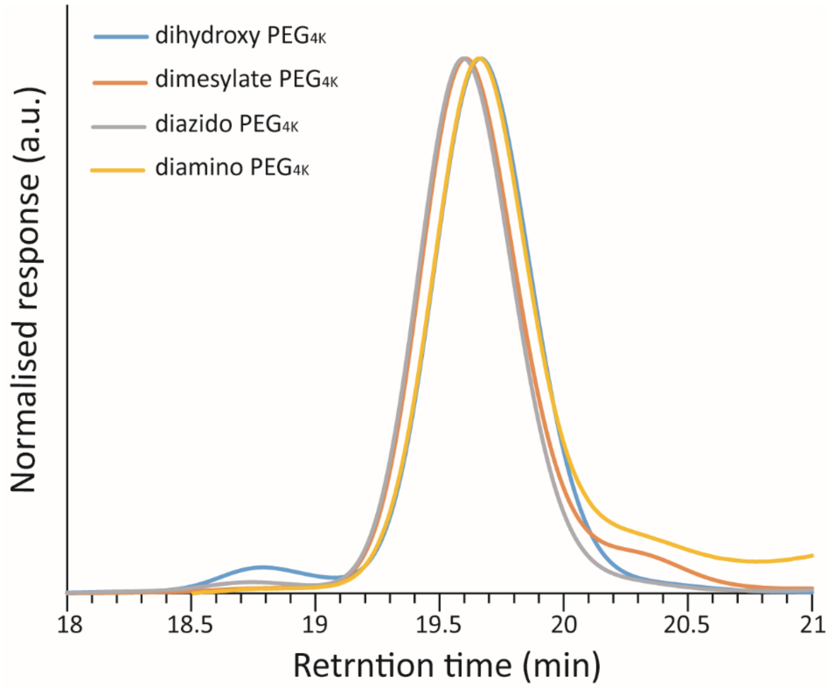

Figure S17. GPC chromatograms showing the conversion of the α,ω-dihydroxy PEG_{4k} to its α,ω-dimesylate, α,ω-diazido and α,ω-diamino PEG_{4k} derivatives recorded using THF as the mobile phase.

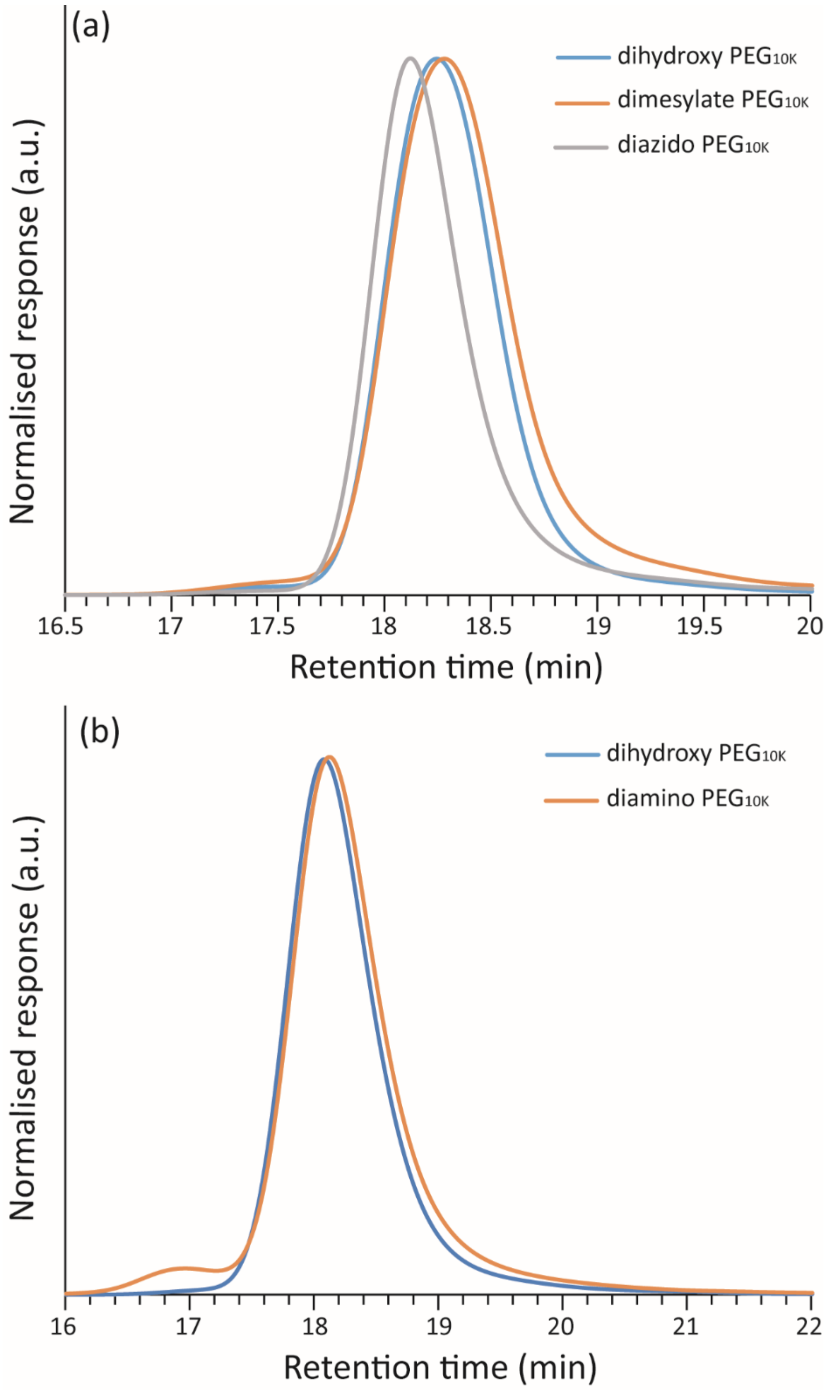

Figure S18. GPC chromatograms showing the conversion of the α,ω-dihydroxy PEG_{10k} to its α,ω-dimesylate, α,ω-diazido and α,ω-diamino PEG_{10k} derivatives recorded using **(a)** THF or **(b)** 1:1 v/v MeOH:water (due to the poor solubility and aggregation of the α,ω-diamino PEG_{10k} derivative in THF) as the mobile phase.

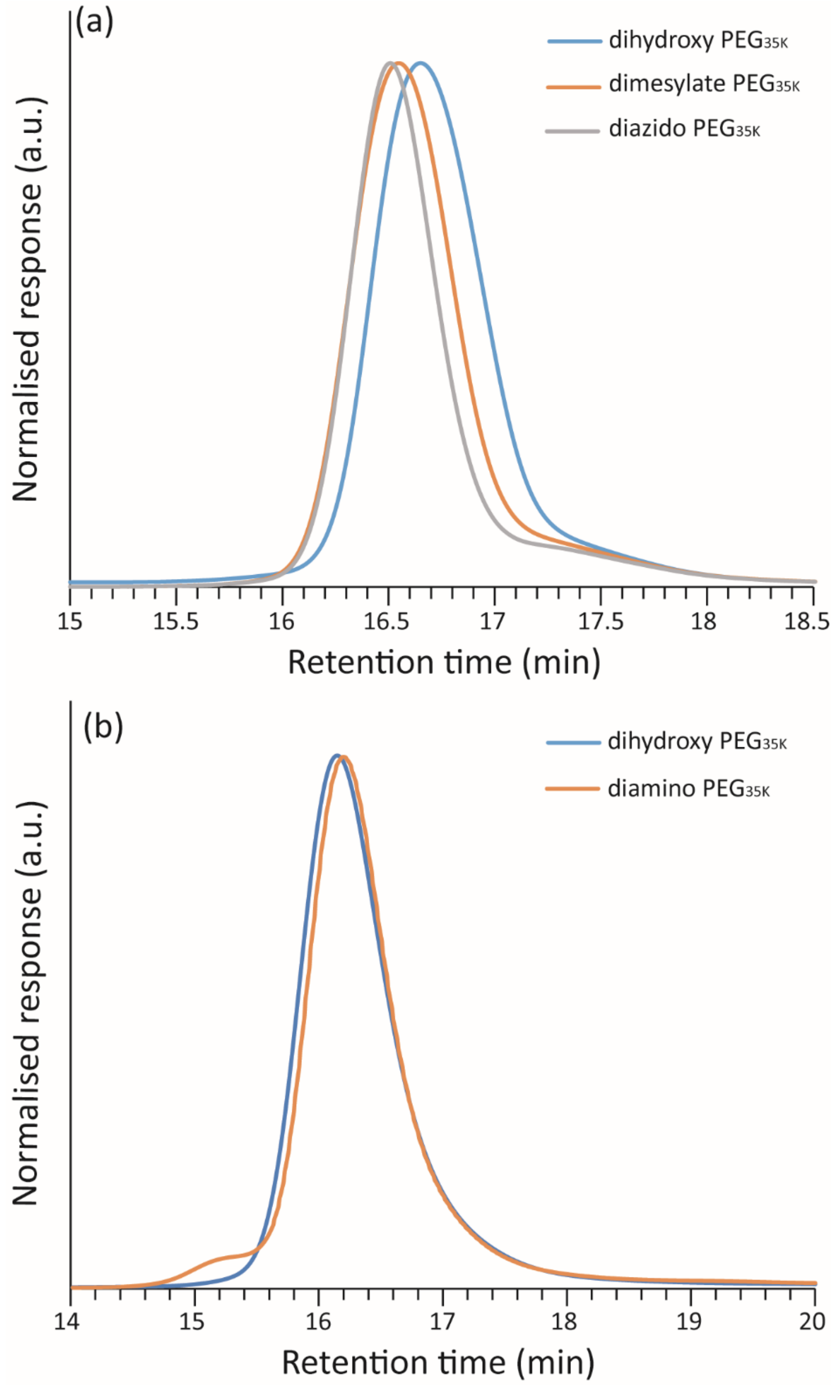

Figure S19. GPC chromatograms showing the conversion of the α,ω-dihydroxy PEG_{35k} to its α,ω-dimesylate, α,ω-diazido and α,ω-diamine PEG_{35k} derivatives recorded using **(a)** THF or **(b)** 1:1 v/v MeOH:water (due to the poor solubility and aggregation of the α,ω-diamino PEG_{10k} derivative in THF) as the mobile phase.

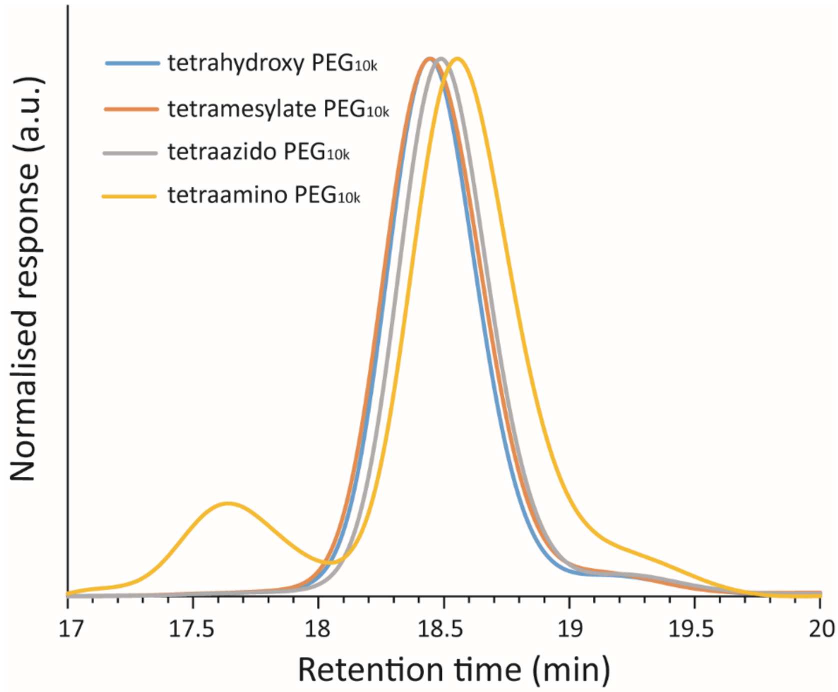

Figure S20. GPC chromatograms showing the conversion of the 4-armed tetrahydroxy PEG_{10k} to its mesylate-, azido- and amino-terminated derivatives recorded using THF as the mobile phase.

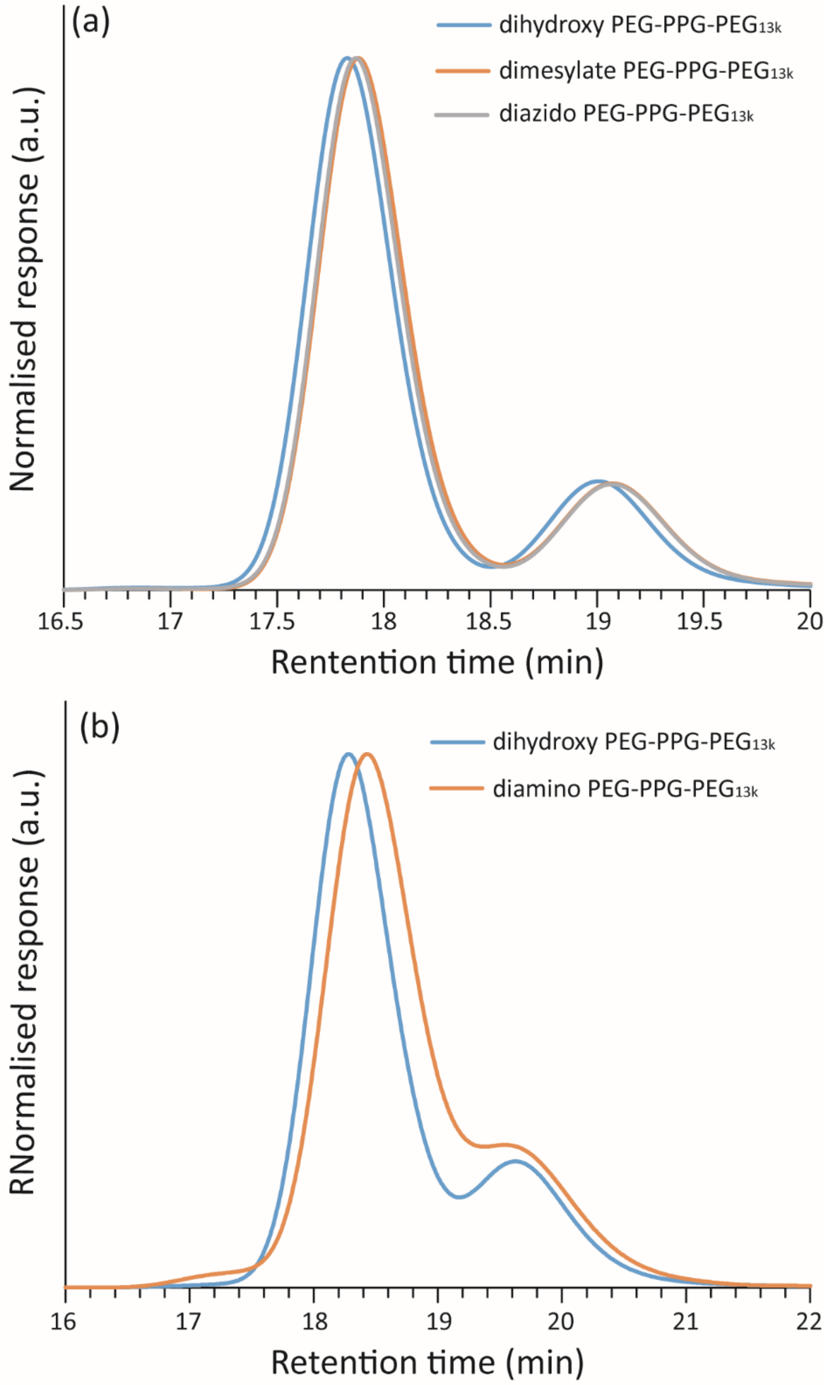

Figure S21. GPC chromatograms showing the conversion of the α,ω-dihydroxy PEG-PPG-PEG (Pluronic® F-127) to its α,ω-dimesylate, α,ω-diazido and α,ω-diamine PEG-PPG-PEG derivatives recorded using **(a)** THF or **(b)** 1:1 v/v MeOH:water (due to the poor solubility and aggregation of the α,ω-diamino PEG_{10k} derivative in THF) as the mobile phase.

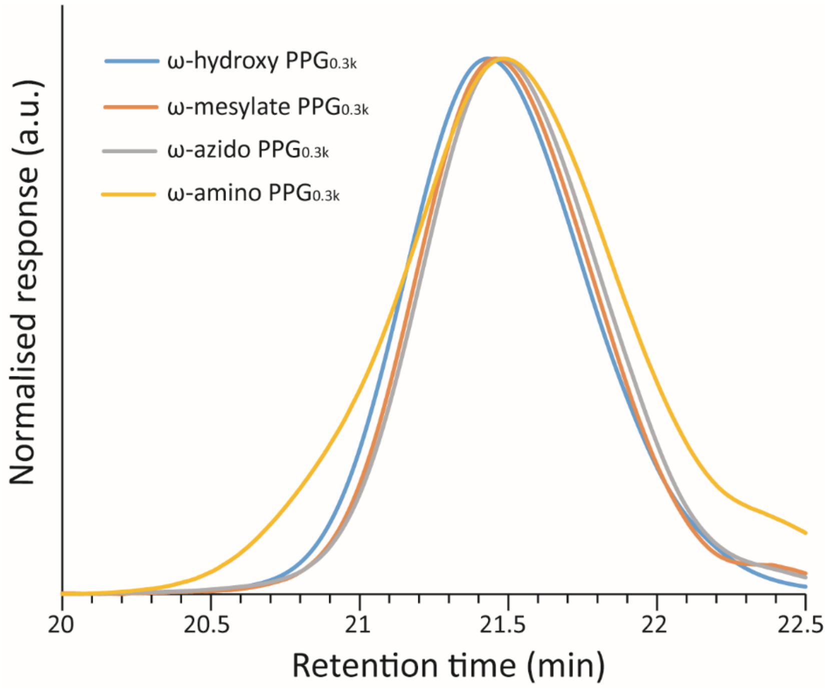

Figure S22. GPC chromatogram showing the conversion of the α-butyl-ω-hydroxy PPG$_{0.3k}$ to its α-butyl-ω-mesylate, α-butyl-ω-azido and α-butyl-ω-amino PEG$_{0.3k}$ derivatives recorded using THF as the mobile phase.

g. Figures for sequential reagent addition synthesis

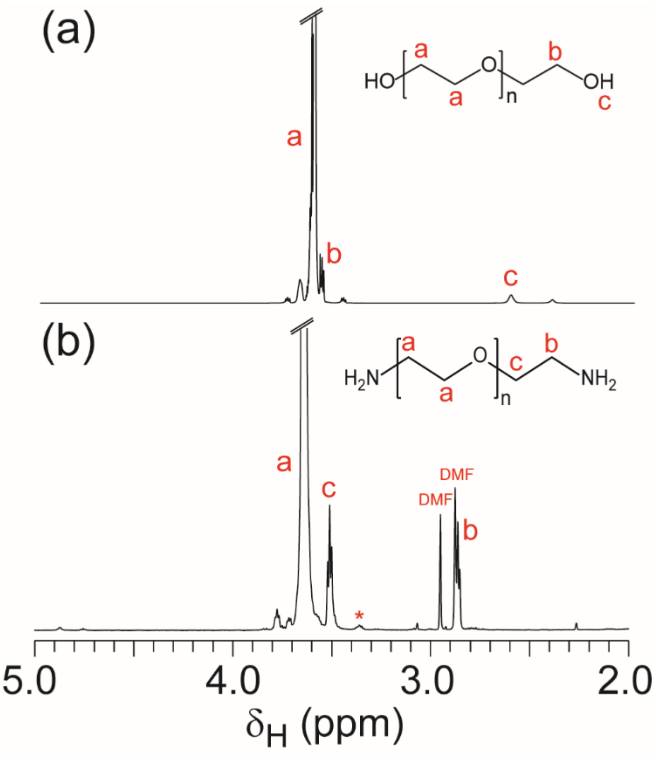

Figure S23. ^{1}H NMR spectra (500 MHz, 23 ℃, $CDCl_3$) of **(a)** α,ω-dihydroxy $PEG_{1.5k}$ and **(b)** the α,ω-diamine $PEG_{1.5k}$ derivative prepared using sequential reagent addition without intermediate purification and isolation steps (* indicates the remaining unreacted azido end-group).

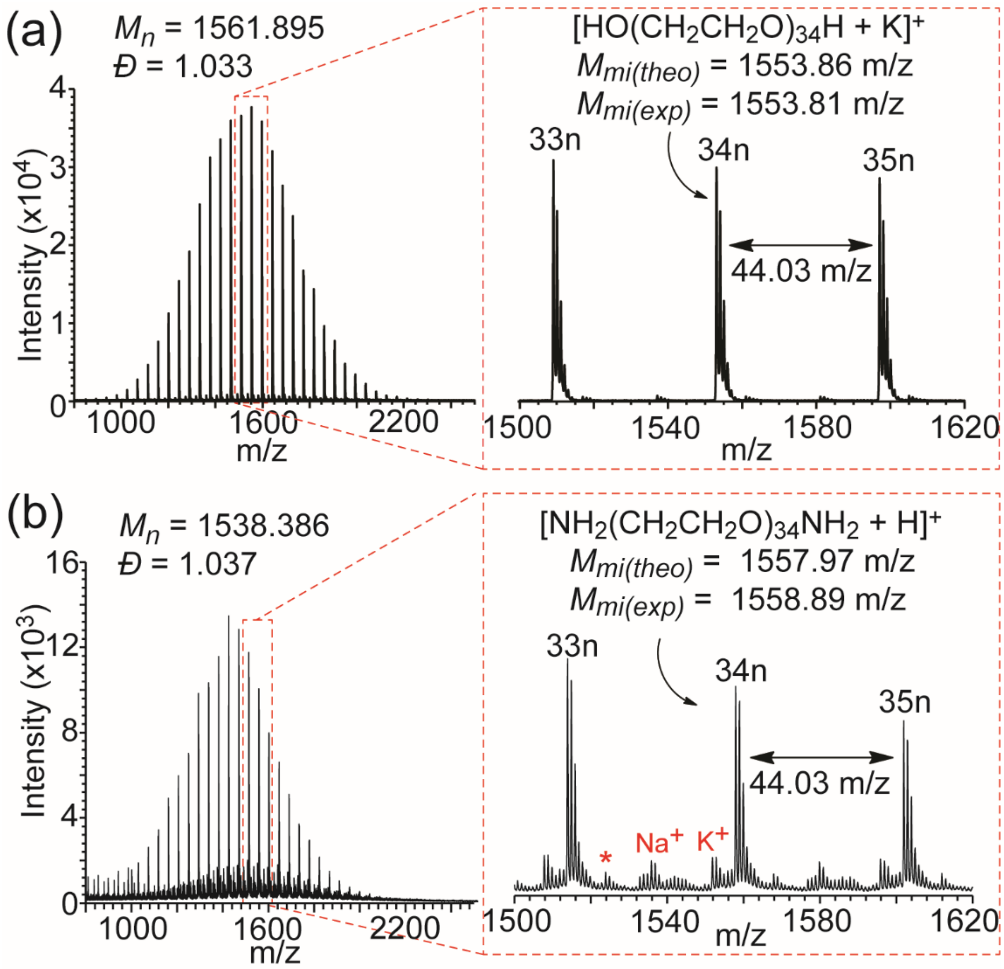

Figure S24. MALDI-ToF mass spectra of **(a)** α,ω-dihydroxy $PEG_{1.5k}$ and **(b)** the α,ω-diamine $PEG_{1.5k}$ derivative prepared using sequential reagent addition without intermediate purification and isolation steps (* indicates the presence of the N_3-PEG-NH_2 series as the proton adduct): expansions show 33-35 repeat unit region of the spectra with the theoretical ($M_{mi(theo)}$) and experimentally determined ($M_{mi(exp)}$) monoisotopic mass values for the potassium or proton adducts of polymers with n = 34.

3. Supporting Table

Table S1. Molecular weight characteristics of the polyglycols determined *via* ^{1}H NMR spectroscopy, MALDI-ToF MS and GPC.

		M_n (kDa)			$Đ$	
**Polymer**	**End-group**	^{1}H NMR[a]	MALDI-ToF MS[b]	GPC[c]	MALDI-ToF MS[b]	GPC[c]
**MeOPEG$_{5.0k}$**	hydroxyl	5.2	5.4	5.4	1.01	1.09
	mesylate	5.2	5.4	5.2	1.01	1.03
	azido	5.2	5.4	5.2	1.01	1.10
	amino	5.2	5.7	5.2	1.02	1.11
**PEG$_{1.5k}$**	hydroxyl	[d]	1.6	1.3	1.03	1.05
	mesylate	1.4	1.7	1.5	1.03	1.06
	azido	1.5	1.6	1.4	1.04	1.09
	amino	1.4	1.6	1.3	1.03	1.06
**PEG$_{4.0k}$**	hydroxyl	[d]	3.4	3.3	1.02	1.07
	mesylate	3.3	3.6	3.3	1.01	1.06
	azido	3.2	3.5	3.4	1.01	1.07
	amino	3.3	3.3	3.1	1.04	1.07
**PEG$_{10k}$**	hydroxyl	[d]	10.2	11.0 (10.9)	1.01	1.08 (1.08)
	mesylate	9.9	10.5	10.4	1.01	1.10
	azido	9.7	10.4	11.9	1.02	1.07
	amino	9.7	10.7	(10.6)	1.01	(1.12)
**PEG$_{35k}$**	hydroxyl	[d]	[e]	33.0 (31.5)	[e]	1.11 (1.14)
	mesylate	31.7	[e]	34.5	[e]	1.11
	azido	31.1	[e]	36.9	[e]	1.10
	amino	31.0	[e]	(31.1)	[e]	(1.17)
**4-Armed PEG$_{10k}$**	hydroxyl	10.6	10.2	9.5	1.01	1.05
	mesylate	10.3	10.3	9.6	1.01	1.05
	azido	10.2	10.1	9.5	1.01	1.02
	amino	10.1	10.1	9.5	1.00	1.11
**Pluronic® F-127**	hydroxyl	[d]	[e]	12.3 (11.9)	[e]	1.22 (1.24)
	mesylate	12.8	[e]	11.5	[e]	1.24
	azido	12.7	[e]	11.7	[e]	1.24
	amino	12.6	[e]	(11.6)	[e]	(1.28)
**^{n}BuOPPG$_{0.3k}$**	hydroxyl	0.5	0.6	0.6	1.08	1.10
	mesylate	0.6	0.6	0.6	1.11	1.08
	azido	0.6	0.5	0.5	1.12	1.10

amino	0.5	0.5	0.5	1.03	1.17

[a] Number-average molecular weight determined from ^1H NMR spectroscopy by comparing the integration of the end-group proton resonances to the repeating unit proton resonances. [b] Number-average molecular weight determined from MALDI ToF MS for the major adduct series, using Polymerix software (Sierra Analytics). [c] Number-average molecular weight determined from GPC versus a conventional column calibration with PEG standards using THF as the mobile phase; values in brackets were determined using 1:1 v/v MeOH:water as the mobile phase. [d] Number-average molecular weight could not be determined from ^1H NMR as the characteristic resonances for the terminal methylene protons adjacent to the hydroxyl groups were not resolved to provide reference for calculation. [e] Number-average molecular weight could not be determined as average mass peaks for the polymer were not resolved.

Reference

1. Zeng, R., Luo, Z., Zhou, D., Cao, F., Wang, Y., A novel PEG coating immobilized onto capillary through polydopamine coating for separation of proteins in CE. *Electrophoresis*, **2010**, *31*, 3334-3341.
2. Zhou, C., Truong, V.X., Qu, Y., Lithgow, T., Fu, G., Forsythe, J.S., Antibacterial poly (ethylene glycol) hydrogels from combined epoxy‐amine and thiol‐ene click reaction. *J. Polym. Sci. Part A: Polym. Chem.*, **2016**, *54*, 656-667.
